# Identification of two different chemosensory pathways in representatives of the genus *Halomonas*

**DOI:** 10.1186/s12864-018-4655-4

**Published:** 2018-04-18

**Authors:** Ana Florencia Gasperotti, María Victoria Revuelta, Claudia Alicia Studdert, María Karina Herrera Seitz

**Affiliations:** 1 Instituto de Investigaciones Biológicas, CONICET - Universidad Nacional de Mar del Plata, Mar del Plata, Argentina; 2 Department of Medicine, Hematology and Oncology Division, Weill Cornell Medicine, New York, NY 10065 USA; 3 Instituto de Agrobiotecnología del Litoral, CONICET - Universidad Nacional del Litoral, Santa Fe, Argentina

**Keywords:** *Halomonas titanicae*, Chemotaxis, Hydrocarbons, C-di-GMP, Diguanylate cyclase

## Abstract

**Background:**

Species of the genus *Halomonas* are salt-tolerant organisms that have a versatile metabolism and can degrade a variety of xenobiotic compounds, utilizing them as their sole carbon source. In this study, we examined the genome of a *Halomonas* isolate from a hydrocarbon-contaminated site to search for chemosensory genes that might be responsible for the observed chemotactic behavior of this organism as well as for other responses to environmental cues.

**Results:**

Using genome-wide comparative tools, our isolate was identified as a strain of *Halomonas titanicae* (strain KHS3), together with two other *Halomonas* strains with available genomes that had not been previously identified at a species level.

The search for the main components of chemosensory pathways resulted in the identification of two clusters of chemosensory genes and a total of twenty-five chemoreceptor genes.

One of the gene clusters is very similar to the *che* cluster from *Escherichia coli* and, presumably, it is responsible for the chemotactic behavior towards a variety of compounds. This gene cluster is present in 47 out of 56 analyzed *Halomonas* strains with available genomes.

A second *che*-like cluster includes a gene coding for a diguanylate cyclase with a phosphotransfer and two receiver domains, as well as a gene coding for a chemoreceptor with a longer cytoplasmic domain than the other twenty-four. This seemingly independent pathway resembles the *wsp* pathway from *Pseudomonas aeruginosa* although it also presents several differences in gene order and domain composition. This second chemosensory gene cluster is only present in a sub-group within the genus *Halomonas*. Moreover, remarkably similar gene clusters are also found in some orders of Proteobacteria phylogenetically more distant from the Oceanospirillales, suggesting the occurrence of lateral transfer events.

**Conclusions:**

Chemosensory pathways were investigated within the genus *Halomonas*. A canonical chemotaxis pathway, controlled by a variable number of chemoreceptors, is widespread among *Halomonas* species. A second chemosensory pathway of unique organization that involves some type of c-di-GMP signaling was found to be present only in one branch of the genus, as well as in other proteobacterial lineages.

**Electronic supplementary material:**

The online version of this article (10.1186/s12864-018-4655-4) contains supplementary material, which is available to authorized users.

## Background

*Halomonas* are Gram negative, motile rods that belong to the family *Halomonadaceae*. Strains belonging to this genus are usually able to adapt to a wide range of salinities, requiring between 2% and 15% NaCl concentration for growth. This feature distinguishes them as useful biotechnological tools, as high salt content greatly reduces sterilization needs. Several *Halomonas* strains have been used in the generation of products of commercial interest as organic osmolytes [1] and different kinds of polyhydroxyalcanoates [2]. Recently, many *Halomonas* strains have been also associated to the degradation of xenobiotic compounds, especially hydrocarbons and aromatic compounds [3, 4], highlighting their potential participation in bioremediation processes, especially in high salt conditions.

Although increasing information is available on *Halomonas* physiology, there are still some features like motility, chemotaxis and chemosensing that remain to be explored.

Chemotaxis is the ability of microorganisms to move towards attractants or away from repellents. This behavior has been studied thoroughly in *E. coli* and *S. typhimurium* and several other bacteria. Briefly, signals are detected by membrane chemoreceptors or MCPs (for Methyl-accepting Chemotaxis Proteins) and transmitted to the flagellar motors through a phosphorylation cascade that controls flagellar rotation and thus the swimming behavior. It has been previously described that chemotaxis represents an advantage for bioremediation processes since it facilitates accessibility to the substrates [5, 6].

All the proteins involved in the chemotaxis-signaling cascade are strongly conserved both in Archaea and Bacteria, and this facilitates the identification of chemosensory systems in the increasing number of bacterial genomes available to date. From the analysis of hundreds of genomic sequences, it became clear that approximately half of the microbial genomes code for multiple chemotaxis-related systems, meaning that they have two or more histidine kinases CheA and several coupling proteins CheW [7]. When multiple chemosensory systems are present, some of them control flagellar motility whereas others control different processes in the cell, like type IV pili-dependent motility, biofilm formation, cell morphology, or cell-to-cell interactions [7]. The number of chemoreceptors coded in the genomes is also very variable, and seems to reflect the complexity of the environment in which the microorganisms live. In contrast to the five MCPs of *E. coli*, many microorganisms have more than ten, and up to fifty-eight MCPs [8].

There is almost no information on the chemotactic behavior of bacteria belonging to the genus *Halomonas*. The strain *Halomonas* sp. KHS3 was recently isolated based on its ability to degrade hydrocarbons and actively swim towards those substrates [9]. The genomic sequence was obtained [10] to get a deeper understanding of the biology of this microorganism. In the present work, the genomic sequence of *Halomonas* sp. KHS3 was examined in search of chemosensory systems. We found two different clusters of chemotaxis-related genes and twenty-five chemoreceptors. One of the gene clusters seems to govern the general chemotactic behavior. The other one, only present in a subgroup of the genus *Halomonas,* codes for a diguanylate cyclase that is presumably controlled by chemosensory stimuli. This second gene cluster displays a novel organization. We found clusters with striking similarities to this one in other proteobacterial species.

This is the first report on chemotaxis-related systems in the genus *Halomonas*, providing the initial step towards experimental studies to deepen our knowledge about the responses of these microorganisms to their chemical environment.

## Methods

### Taxonomic analyses

#### Average nucleotide identity ANI

ANI values were calculated using the BLAST-based ANI calculation method (ANIb) [11, 12]; ANI values between genomes of the same species are above 95%. Results were obtained from JSpecies Web Server (http://jspecies.ribohost.com/jspeciesws).

#### Tetranucleotide frequency correlation coefficients

The TETRA web-service computes correlation coefficients between tetranucleotide usage patterns of DNA sequences, which can be used as an indicator of sequence relatedness [12]. *Halomonas* sp. KHS3 was taken as reference. Results were obtained from JSpecies Web Server (http://jspecies.ribohost.com/jspeciesws) [12].

#### Genome-to-genome distance calculation (GGDC)

This tool provides similarity values analogous to DNA-DNA Hybridization (DDH). Distances are inferred using three distinct formulas from the set of HSPs (**H**igh **S**core **P**airs) obtained by comparing each pair of genomes with the chosen software. These distances are transformed to values analogous to DDH (estimated DDH) using a generalized linear model (GLM) inferred from an empirical reference dataset comprising real DDH values and genome sequences [13]. Results were obtained from DSMZ web server (http://ggdc.dsmz.de/distcalc2.php).

Draft genomic sequences of *Halomonas* sp. KHS3 [10] and from other 56 *Halomonas* strains (Additional file 1: Table S1) were obtained from NCBI database, Ensemble Bacteria and PATRIC.

### Transmission Electron microscopy imaging

Negative staining of cells for TEM imaging was carried out as follows:

A droplet of the cell suspension was placed onto a copper grid (400 mesh), covered with collodion during 5 min. The excess of collodion was drained with filter paper. Cells adhered to the grids were contrasted with 2% phosphotungstic acid for 40 s. Samples were examined with a transmission electron microscopy, JEM 1200 EX II (JEOL Ltd., Tokyo, Japan) and pictures taken with a Erlangshen ES1000W, Model 785 camera (Gatan Inc., Pleasanton, California, USA) in the Central Electronic Microscopy Service of Veterinary Sciences Faculty, Universidad Nacional de La Plata, Argentina.

### Identification of chemosensory systems in *H.titanicae* KHS3

The draft genome sequence of *Halomonas sp.* KHS3 [10] was analyzed using the RAST Annotation Server [14] and the Integrated Microbial Genomes Database [15]. All the genes found in the *Halomonas* sp. KHS3 genome that coded putative chemosensory related proteins were compared to the well described *E. coli* chemotaxis proteins (CheA, CheW, CheR, CheB, CheY, CheZ and MCPs) using NCBI available sequences and Clustal Omega or MAFFT (EMBL- EBI) for the alignments. Protein domains were identified using the InterPro Scanning tool [16] and NCBI structure tool (http://www.ncbi.nlm.nih.gov/Structure/cdd/wrpsb.cgi).

Structure modeling was done in Phyre2 [17] and SWISS-MODEL servers [18]. Tetratricopeptide repeats were analyzed using Bioinformatic Toolkit (Max-Planck Institute for developmental Biology, https://toolkit.tuebingen.mpg.de/tprpred) [19].

Operon prediction was based on directions of adjacent genes, distribution of intergenic distances and presence of predicted promoter regions using FGENESB (Softberry) [20].

### Phylogenetic trees

16S and 23S data were retrieved from the available *Halomonas* sp. genomes and aligned with SINA (version 1.2.11) [21], specifying SSU or LSU. The aligned 16S-23S rRNA sequence files in nexus format were annotated with the BEAuti software in the BEAST package (version 2.4.5) [22] with the Gamma site model and estimated substitution rates. The phylogenetic relationships of the 16S-23S merged alignments were inferred using the MCMC Bayesian algorithm implemented in BEAST. BEAST analyses were run twice, with two independent runs, with 1 × 10^7 iterations per run, sampling every 1000 steps. The resulting runs were inspected with Tracer and the maximum clade credibility tree was searched using the LogCombiner and TreeAnnotator software with a 20% burn in. The resulting tree converged with all nodes supported with a posterior probability > 0.5.

*Halomonas* sp. genomes (Additional file 1: Table S1), Swissprot and PDB were scanned with a cheA query using Phmmer [23] in an iterative approach. Resulting sequences were aligned with MAFFT 7 [24] and manually inspected. Phylogenetic analyses were performed with the maximum likelihood package PhyML3 [25]. All sequences were scanned for motifs in http://www.genome.jp/tools/motif/ using PROSITE and PFAM as databases.

Trees were visualized with Dendroscope v3.2.10 [26] and final tree editing was done using iTOL [27].

### Chemotaxis assays

For the screening of general chemotactic behavior of *H. titanicae* KHS3 soft agar plates were prepared with modified H1 minimal medium [28], 0.3% agar, with neither thiamine nor supplementary amino acids, supplemented with 2% (*w*/w) NaCl to allow optimal growth of *H. titanicae* KHS3. Different carbon sources were added to a 25 mM final concentration with the exception of sodium salicylate, phenanthrene and phthalate that were used to 50 μg ml^− 1^ final concentration. Plates were inoculated with fresh colonies of *H. titanicae* KHS3 and incubated 24 – 48 h at 30 °C.

## Results

### Taxonomical identification of *Halomonas* sp. KHS3 strain

*Halomonas* sp. KHS3 was isolated from seawater of Mar del Plata harbor, a hydrocarbon-contaminated site, based on its ability to grow and show chemotactic responses towards hydrocarbons [9]. It belongs to the Class of the *Gammaproteobacteria*, Order Oceanospirillales, Family *Halomonadaceae*. Although the taxonomy of this family is under continuous revision at least 15 genera have been described (https://www.arb-silva.de/browser/), the genus *Halomonas* being one of them, with 96 species reported to the moment of writing (http://www.bacterio.net/halomonadaceae.html).

Strain KHS3 was initially identified as *Halomonas* sp. based on a BLAST alignment of its RNA 16S sequence that showed 99% of similarity to *Halomonas titanicae* strain S6-2-2 and *Halomonas* sp. MBEE15 [9].

The genomic sequence of *Halomonas* sp. KHS3 was recently obtained and annotated by our group [10]. To get an accurate taxonomic identification of *Halomonas* sp. KHS3, three different bioinformatic tools were used to compare the whole genomic sequence of *Halomonas* sp. KHS3 with those of other *Halomonas* species sequenced up to date. Average Nucleotide Identity was calculated against all the *Halomonas* species with available genome sequences. The highest similarity values were obtained with *Halomonas* sp.19A GOM-1509 m and *Halomonas titanicae* BH1, with a nucleotide identity of 97.52% and 97.22% respectively. Likewise, the Genome-to-Genome Distance Calculation (GGDC) gave an estimated DDH value (DNA-DNA hybridization, *see* *Methods*) of 79.9% between *Halomonas sp.* KHS3 and *H. titanicae* BH1. Furthermore, the tetranucleotide correlation coefficient obtained between these two strains was also very close to 1 (0.99957). As microorganisms belonging to the same species give ANI values higher than 95% [11] and DNA-DNA hybridization values higher than 70% [13], *Halomonas sp.* KHS3 was identified as a strain of *H. titanicae*. Table 1 summarizes the values obtained from the comparison between *H. titanicae* KHS3 and the most closely related species of the genus, as well as with the type strain *H. elongata* DSM 2581. Values obtained for two other strains*, Halomonas* sp. 19A GOM-1509 m and *Halomonas* sp. A3H3, indicate that they are also strains of *Halomonas titanicae*.

**Table 1:** Genomic comparison between *Halomonas* sp. KHS3 and other *Halomonas* species

Microorganism	ANI^a^	Tetranucleotide signature Z score^b^	GGDC (%)^c^
***Halomonas*** **sp. KHS3**	100	1	100
***Halomonas*** **sp. 19A GOM-1509 m**	97.52	0.9997	82.20
***Halomonas titanicae*** **BH1**	97.22	0.99957	79.90
***Halomonas*** **sp. A3H3**	96.25	0.9991	73.80
*Halomonas* sp. R57-5	87.54	0.99779	34.90
*Halomonas* sp. TG39a	87.40	0.99769	35.00
*Halomonas boliviensis* LC1	86.60	0.99602	33.30
*Halomonas* sp. KO116	85.23	0.99569	30.80
*Halomonas elongata* type strain DSM 2581	71.00	0.66802	20.10

Microorganisms in bold letter indicate *Halomonas titanicae* strains

^a^Average Nucleotide Identity

^b^Analysis of tetranucleotide frequencies

^c^Genome to Genome Distance Calculator: DNA-DNA hybridization estimate

### Motility and chemotactic behavior of *H. titanicae* KHS3

**Fig. 1:**
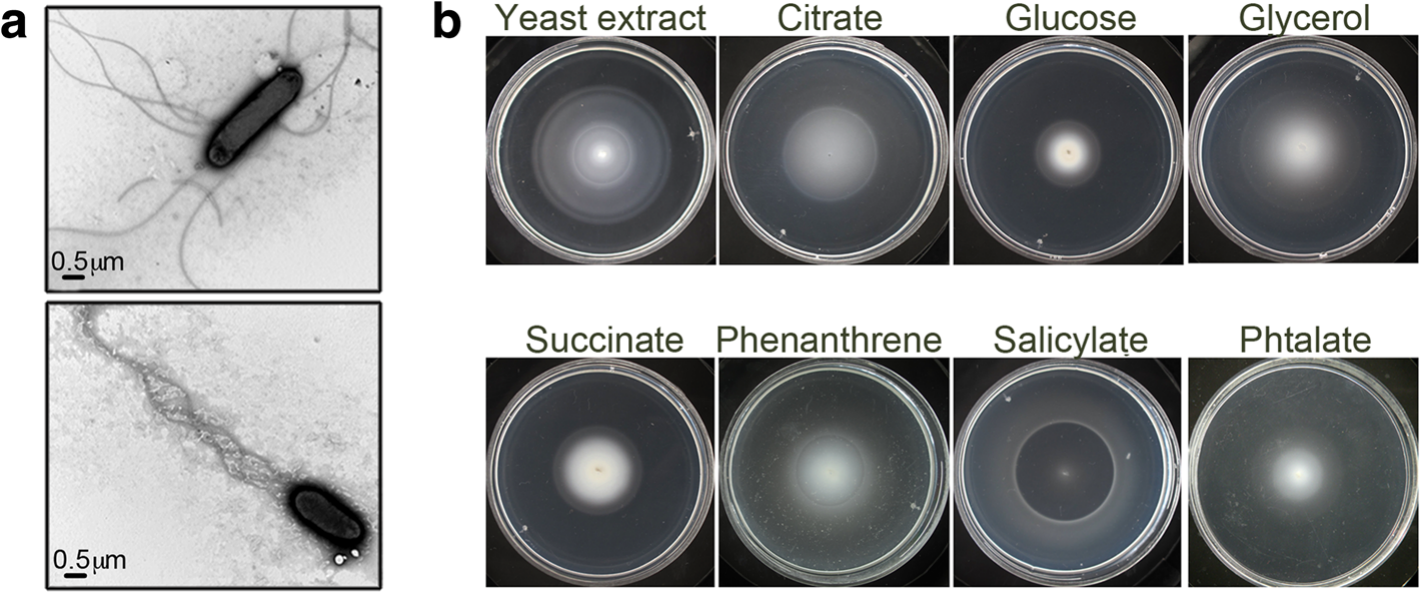
Chemotactic behavior of *Halomonas titanicae* KHS3. **a** TEM images of *H. titanicae* KHS3. Cells were negatively stained as indicated in *Methods*. Two different cells obtained from the same culture are shown. **b** Minimal medium H1 soft-agar plates containing different carbon and energy sources (as indicated) were prepared as described in *Methods*. Bacteria were inoculated in the center of the plate and incubated at 28-30 °C for 24-48 h

*H. titanicae* KHS3 looks highly motile under the microscope in liquid cultures. Transmission electron microscopy images showed peritrichous flagella (Fig. 1a) as has also been reported for *H. titanicae* BH1 [29].

The chemotactic behavior of *H. titanicae* KHS3 towards different carbon and energy sources was analyzed in minimal medium soft-agar plates with the indicated carbon sources. In these plates, bacteria create chemical gradients upon substrate consumption and display macroscopically visible chemotactic rings as they follow those gradients in a coordinated fashion. *H. titanicae* KHS3 gave clear chemotactic responses towards sugars as glucose (Fig. 1b) and maltose (Additional file 2: Figure S1) and also to organic acids like citrate, succinate (Fig. 1b), lactate and malate (Additional file 2: Figure S1). A chemotaxis defective mutant derivative isolated in our laboratory was unable to develop those rings, in spite of showing nomal motility and growth in the tested carbon sources (Additional file 2: Figure S1). Since *H. titanicae* KHS3 was isolated based on its ability to show chemotactic responses to gasoil [9] different compounds related with hydrocarbon metabolism were added as the sole carbon and energy source in minimal medium soft agar-plates. Chemotaxis rings were observed after 24-48 h incubation in plates containing phenanthrene as well as in plates containing sodium salicylate or phtalate (Fig. 1b), indicating that *H. titanicae* KHS3 is chemotactic not only to phenanthrene but also to intermediate degradation compounds.

Together, these results show that *H. titanicae* KHS3, as many other environmental microorganisms, is able to sense a wide variety of compounds.

To determine whether the ability to sense and degrade polyaromatic hydrocarbons was a strain-specific characteristic of our isolate, we assessed these abilities in the reference strain *H. titanicae* BH1. The strain BH1 was able to grow with phenanthrene as the sole carbon source and showed a chemotactic response to this hydrocarbon comparable to the one observed for the strain KHS3, as well as towards different substrates (Additional file 2: Figure S1).

### Identification of chemotaxis-related genes in the genomic sequence of *H. titanicae* KHS3

**Fig. 2:**
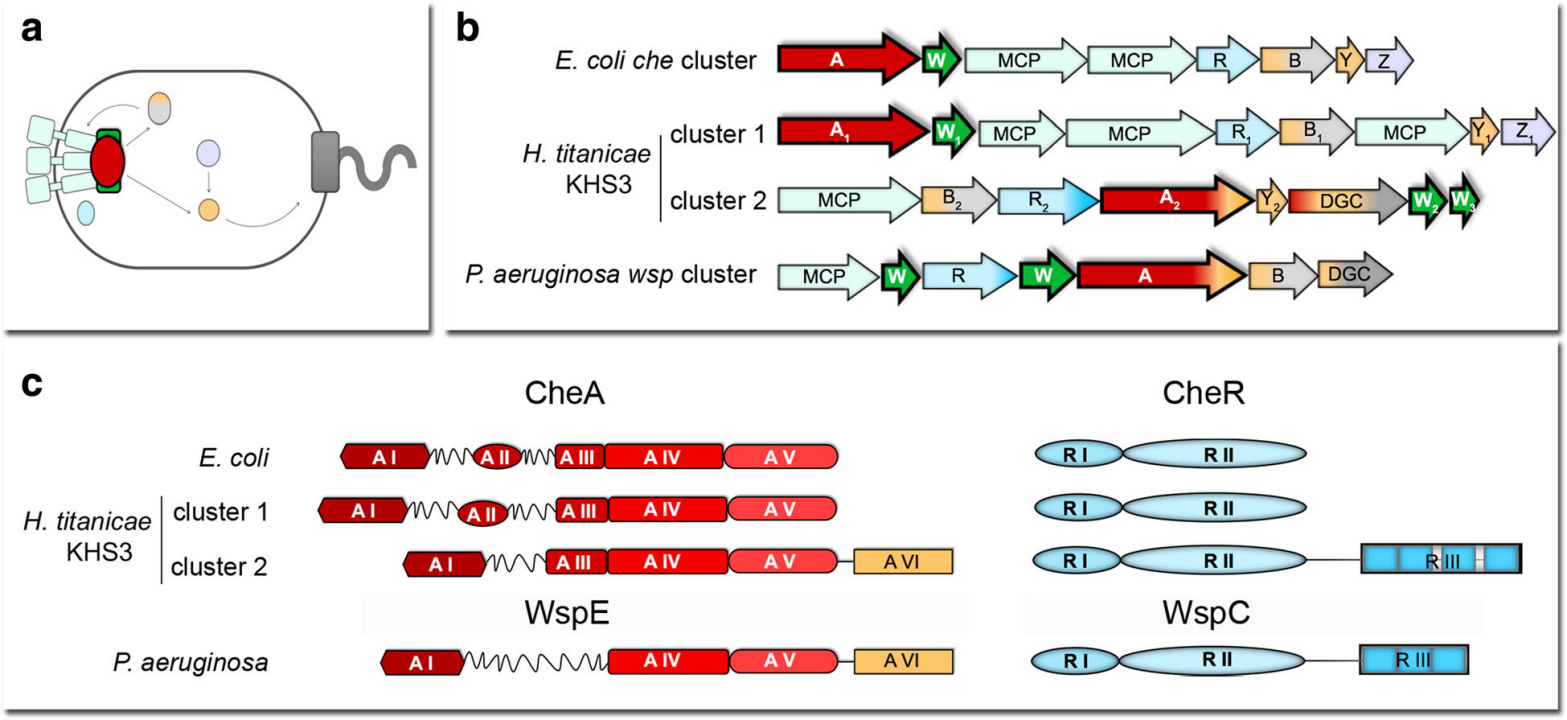
Chemosensory clusters identified in the genomic sequence of *H. titanicae* KHS3. **a** Schematic representation of the localization and interactions of *E.coli* chemotaxis proteins. Light green, chemoreceptors; red oval, CheA; dark green square, CheW; lilac, CheZ; light blue oval, CheR; light gray, methylesterase domain from CheB; light orange, receiver domains/proteins (CheB and CheY); dark gray square, flagellar motor. **b** Gene organization of chemosensory clusters in *E. coli*, *H. titanicae* KHS3 and *P. aeruginosa* (cluster *wsp*). Each gene is represented as an arrow. Light green, chemoreceptors; red, cheA; dark green, cheW; lilac, cheZ; light blue cheR; light gray, cheB; light orange, receiver domains (in CheB, diguanylate cyclase, CheA and CheY); dark gray, diguanylate cyclase domain. MCPs in *H. titanicae* KHS3 cluster 1 are RO22_21455, RO22_21470 and RO22_21475. The only MCP in cluster 2 is RO22_21155. **c** Domain organization of CheA and CheR proteins; A I: Hpt or histidine phosphotransfer domain (P1), A II: CheY/B binding domain (P2), A III: signal transducing histidine kinase, homodimerization domain (P3), A IV: HATPase histidine kinases like ATPase domain (P4), A V: CheW-like domain (P5), A VI, response regulator. R I, methyltransferase domain; R II, S-adenosyl methionine binding; R III, tetratricopeptide repeats

Unlike enteric bacteria, that possess a single set of genes involved in chemotactic behavior, many environmental strains carry several chemotaxis-related genes that have been implicated in chemotaxis or alternative cellular functions [7]. The genomic sequence of *H. titanicae* KHS3 was analyzed to find out potential chemosensory systems using the annotation of *H. titanicae* KHS3 genome in RAST and the JGI Integrated Microbial Genomes servers. The search for the two central components of any chemosensory transduction pathway, namely the histidine kinase CheA and the coupling protein CheW, led to the identification of two different clusters of chemosensory genes (Fig. 2b).

#### Chemosensory cluster 1

This gene cluster contains a set of genes typically present in chemotaxis clusters. Besides genes coding for the histidine kinase CheA (CheA1) and the coupling protein CheW (CheW1), it contains genes coding for homologs of the methyltransferase CheR (CheR1), the methylesterase CheB (CheB1), the single domain response regulator CheY (CheY1) and the phosphatase CheZ (CheZ1), together with three chemoreceptor genes (Fig. 2b). All the predicted proteins coded in cluster 1 are highly similar to their *E. coli* homologs with protein identity values higher than 55% (Additional file 3: Table S2). Furthermore, the organization of genes in cluster 1 is almost identical to that observed in *E. coli che* cluster (Fig. 2b).

As in *E. coli*, genes coding for flagellar proteins were found nearby, also close to a gene that codifies for an RNA polymerase alternative sigma factor, σ^28^. A search for promoter-like sequences revealed several putative σ^28^ binding sites upstream of cluster 1 genes, suggesting that genes from cluster 1 might also share regulatory features with chemotaxis genes from enteric bacteria and *Bacillus subtilis*, that are under the control of σ^28^ [30].

#### Chemosensory cluster 2

This second cluster includes genes coding for a chemoreceptor, CheB2, CheR2, CheA2, CheY2, a protein with a GGDEF diguanylate cyclase domain and finally CheW2 (Fig. 2b) followed by a small hypothetical protein that in a deeper analysis (*see below “Analysis of the main components…”*) also displays structural homology to CheW and was consequently named CheW3. All these che-like genes are much more distantly related to their homologs in *E. coli* than the ones from chemosensory cluster 1 (Identity percentages ranging between 17 and 35%; Additional file 3: Table S2). The organization of genes and the absence of intergenic sequences in cluster 2 suggest that it constitutes an operon. Cluster 2 strongly resembles the previously described *wsp* cluster from *Pseudomonas fluorescens* [31] and *P. aeruginosa* [32], which codes for closely related che-like proteins but has been shown to be involved in biofilm formation and not in chemotaxis [31, 33]. As in *wsp* cluster, cluster 2 includes a gene coding for a protein containing a diguanylate cyclase (DGC) domain whose activity is presumably controlled by receiver domains, and other chemotaxis-related genes that differ from the canonical ones both in sequence and in domain organization (Fig. 2b, c). However, several differences exist between chemosensory cluster 2 and cluster *wsp.* As can be seen in Fig. 2b, the gene order is clearly different, and there are also some differences in gene composition. Whereas cluster 2 codes for a CheY-like protein, this gene is absent in cluster *wsp*. Besides, there are some differences in the domain composition of individual predicted proteins.

No flagellar related genes were found close to the non-canonical cluster 2, reinforcing the idea that cluster 2 might be involved in the control of processes not related to flagella-mediated motility.

#### Chemoreceptors

In total, 25 chemoreceptor genes were identified in the genome of *H. titanicae* KHS3. Three MCPs are included in chemosensory cluster 1 (RO22_21455, RO22_21470 and RO22_21475) and one in chemosensory cluster 2 (RO22_21155) (Fig. 2b), whereas the other 21 MCPs are spread in the genome.

Chemoreceptors are homodimeric proteins usually bound to the cytoplasmic membrane, in most cases carrying a periplasmic ligand-binding domain (LBD) flanked by two transmembrane segments. Following the second transmembrane segment most chemoreceptors have HAMP domains (commonly found in **H**istidine kinases, **A**denylate cyclases, **M**ethyl-accepting proteins and **P**hosphatases) that consist in a parallel α-helical bundle of approximately fifty residues [34]. After one or more HAMP domains follows the highly conserved cytoplasmic region. This consists of a long coiled-coil hairpin including a membrane distal signaling region, where interaction both between chemoreceptors and with CheA and CheW takes place, and a membrane proximal methylation region including the residues responsible for adaptation to stimuli. Even though the signaling region is strongly conserved within Eubacteria and Archaea, the overall length of the hairpin varies due to symmetric seven-residue (heptad) insertions or deletions that have appeared over the course of evolution. Thus, chemoreceptors can be classified in families or classes according to the number of heptads present in the cytoplasmic hairpin [35].

**Fig. 3:**
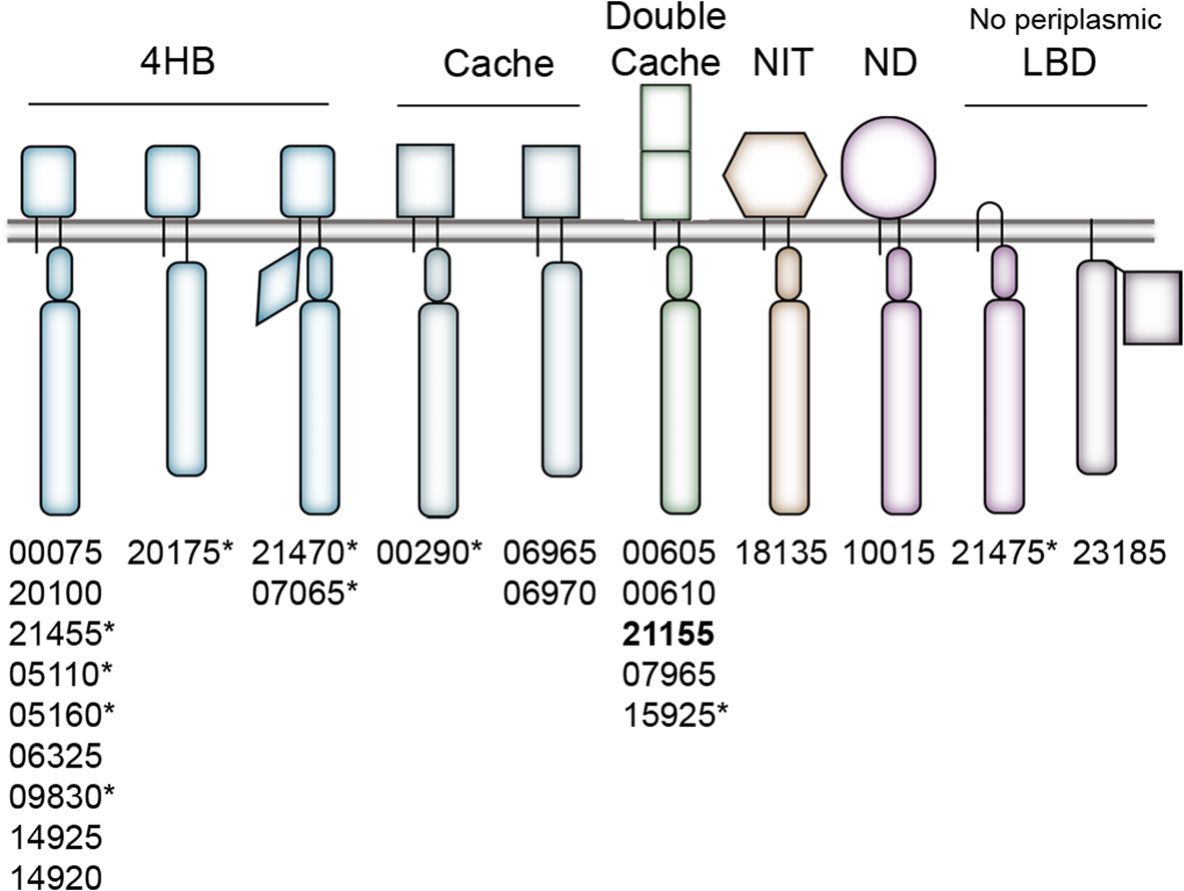
Schematic representation of chemoreceptors encoded in the genome of *H. titanicae* KHS3. MCPs are grouped according to the predicted structure of the periplasmic LBD: 4-helix bundle (4HB, rectangle with curved edges), Cache (rectangle), double Cache (double rectangle), nitrate-nitrite sensing fold (NIT, hexagon), not determined (ND, circle) and those with no periplasmic LBD. The gray horizontal bar represents the cytoplasmic membrane. MCP cytoplasmic subdomains are represented by a long rectangle (conserved cytoplasmic domain or signaling domain), an oval representing the HAMP domain, a diamond shape representing PAS domain. The rectangle at the C-terminus of RO22_23185 represents a Cache domain. The corresponding MCP ID numbers are listed below each kind of MCP (all ID numbers should be preceded by “RO22_” following the IMG gene annotation). Asterisks indicate the presence of C-terminal pentapeptide for interaction with CheR. All MCPs belong to the 36H family except one, which belongs to the 40H family (shown in bold)

The general architecture of the predicted MCPs is shown in Fig. 3. All 25 predicted MCP genes have at least one transmembrane segment and 23 out of the 25 MCPs contain a predicted periplasmic LBD. An analysis of the predicted structures of all the periplasmic LBDs using the Phyre2 server shows that most of them display the structures commonly found among chemoreceptors: 4HB for four-helix bundle (thirteen), Cache (three) or double Cache (five). One LBD shows a NIT (nitrate- and nitrite-sensing)-like folding, suggesting that it might sense nitrogen compounds [36]. The periplasmic LBD from RO22_10015 does not fit to any known structure. RO22_21475 has only a short periplasmic region, with no identifiable LBD domain, and RO22_23185 completely lacks a periplasmic domain but carries an extra domain after the signaling hairpin that showed a DcuS-like structure (Cache domain) when modeled with Phyre2 server.

Of the 25 MCPs identified in *H. titanicae* KHS3, 24 belong to the 36H family according to the classification of Alexander and Zhulin [35], meaning that their cytoplasmic domains have 36 heptads. Of these MCPs, eleven carry at their C-terminus the pentapeptide that is presumably important for methyltransferase recruitment into the chemoreceptor complex for efficient adaptation (Fig. 3) [37]. The MCP coded in cluster 2 belongs to a different length class (40H), suggesting that it assembles and signals independently [38] to control the activity of the cluster 2 proteins.

Although for most MCPs it was not possible to assess their putative function based on their genomic context, in some cases there are some hints. Thus, we found MCP genes close to genes related to arsenic resistance (RO22_20100), to genes related to amino acid binding or metabolism (RO22_10015, RO22_07065 and RO22_07070) or to the degradation of heterocyclic aromatic compounds (RO22_15925).

Work in progress in our laboratory is aimed to elucidate the pattern of ligands for each of the MCPs.

### Analysis of the main components coded in chemosensory clusters

In this section we describe in more detail those proteins that show major differences between the two chemosensory clusters.

#### CheA

The histidine kinase from *H. titanicae* cluster 1 contains the same five domains as the canonical *E.coli* CheA [P1 (Hpt or histidine phosphotransfer domain, A I in Fig. 2c), P2 (CheY/B binding domain, A II in Fig. 2c), P3 (signal transducing histidine kinase, homodimerization domain, A III in Fig. 2c), P4 (HATPase histidine kinases like ATPase domain, A IV in Fig. 2c) and P5 (CheW-like domain, A V in Fig. 2c)]. In contrast, the histidine kinases coded both in chemosensory cluster 2 from *H. titanicae* (CheA2) and in cluster *wsp* (WspE) from *P. aeruginosa* lack the response regulator-binding domain and have an additional receiver domain at their C-terminus (A VI in Fig. 2c, left). In a similar protein from *Myxococcus xanthus* (FrzE), the additional receiver domain works as a negative regulator for the autophosphorylation of the kinase [39] but there are not many examples of other CheA/Y proteins characterized.

#### CheR

**Fig. 4:**
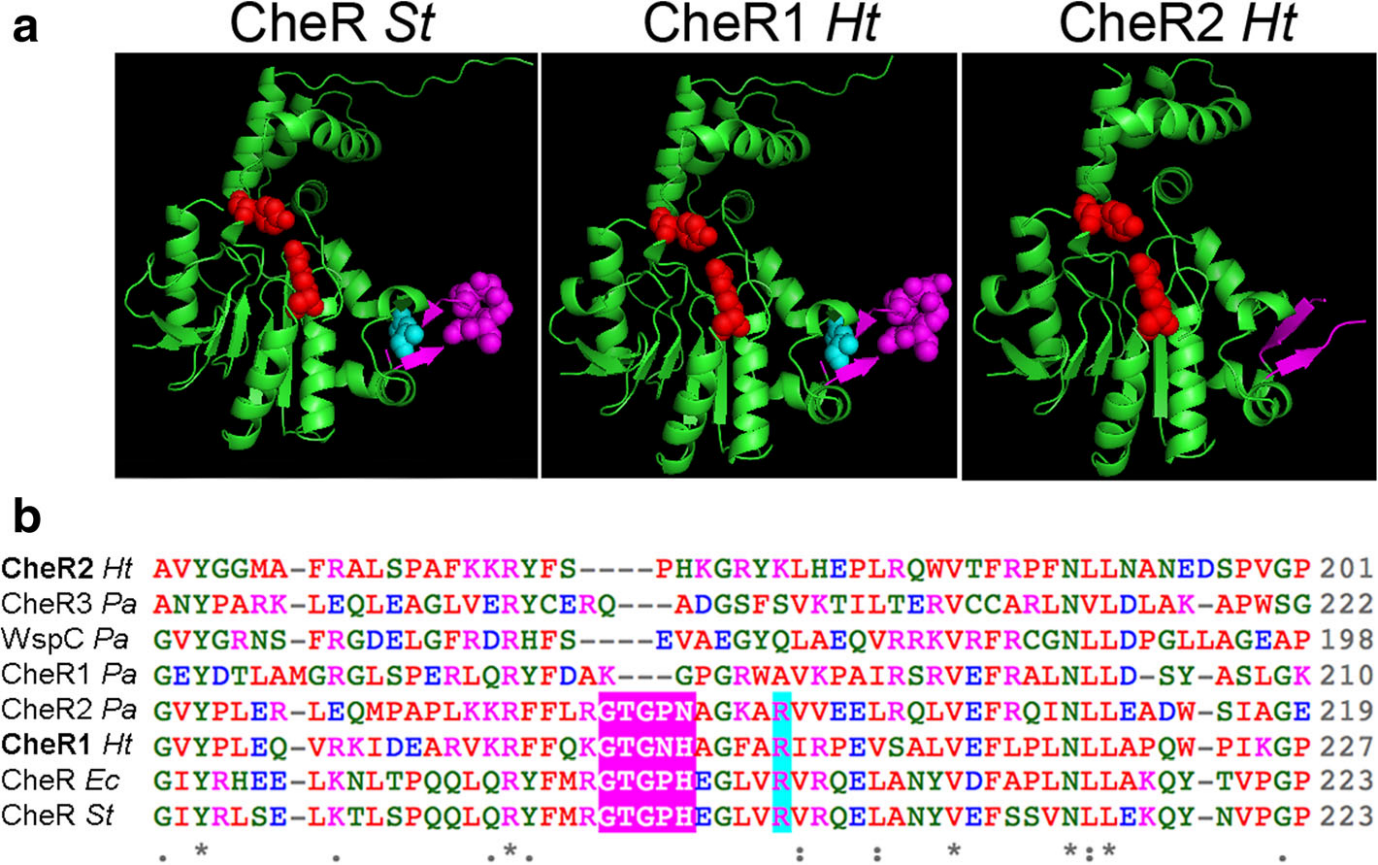
Predicted structure of CheR1 and CheR2 from *H. titanicae* KHS3. Amino acid sequences of both cheRs were modeled using Phyre2 server. **a** Comparison between the template and models. Structure of the template CheR from *S. typhimurium* (PDB accession code 1af7, left panel) and the models for CheR1 *Ht* (middle panel) and CheR2 *Ht* (right panel). Residues R98 and Y235 from CheR *St* or the equivalent positions in the models are shown as red spheres (see full alignment in Additional file 2: Figure S1). The β-subdomain that contains the loop responsible for interaction with chemoreceptor pentapeptides is colored fuchsia and the critical residue for this interaction (R197 in CheR *St*) is colored cyan. **b** Portion of the amino acid sequence alignment from CheR *St* (P07801), CheR *Ec* (P07364), CheR1 *Pa* (PA3348), CheR2 *Pa* (PA0175) and CheR3 *Pa* (PA0412), CheR1 *Ht* (RO22_21465) and CheR2 *Ht* (RO22_21165). Residues that constitute the β-loop responsible for interaction with chemoreceptor pentapeptides are highlighted fuchsia. Residue R197 from CheR *St* and its equivalents are highlighted cyan. For a complete alignment see Additional file 2: Figure S1

An alignment between the CheR protein sequences from *E. coli, S. typhimurium*, *P. aeruginosa* (three genes from different clusters) and *H. titanicae* KHS3 (two genes from clusters 1 and 2) shows that all of them contain conserved residues that have been shown to be important in the active site region of CheR from *S. typhimurium* as R98, D154 and Y235 [40, 37] (Additional file 4: Figure S2), suggesting that both CheR1 and CheR2 are active enzymes. However, they show differences in the length of the β-loop involved in the recognition of the C-terminal pentapeptide tether that is present in certain chemoreceptors [41] (Fig. 4a, b). In the alignment, it is clear that this loop is longer in *S. typhimurium* and *E. coli* CheRs and also in *P. aeruginosa* CheR2, being all of them enzymes known to bind to C-terminal pentapeptides in chemoreceptors [37, 41]. Besides, these same three CheRs also carry the conserved residue R197 that was identified as responsible for the interaction with the tryptophan residue in the C-terminal pentapeptide (Fig. 4b) [37]. Only CheR1, the putative canonical CheR from *H. titanicae*, shows a good alignment in this β-loop region, while CheR2 from cluster 2 contains a shorter loop and lacks the residue R197 (Fig. 4a, b). This suggests that the canonical CheR1 is responsible for the methylation of the eleven pentapeptide-containing chemoreceptors from *H. titanicae* KHS3, and presumably also for the methylation of all the other chemoreceptors that belong to the same class and, most likely, form part of the same chemoreceptor array.

On the other hand, similar to WspC, the CheR coded in cluster *wsp* [42], the methyltransferase coded in chemosensory cluster 2 (CheR2) contains an additional domain at the C–terminus identified as tetratricopeptide repeat (TPR) with four repeats (Fig. 2c, right; Additional file 4: Figure S2). In WspC from *P. aeruginosa,* the deletion of the TPR domain seems to abolish substrate methylation [33]. In FrzF, another CheR-TPR protein from *M. xanthus,* the removal of its TPR domain causes a change in its methylation specificity [43].

#### CheW

**Fig. 5:**
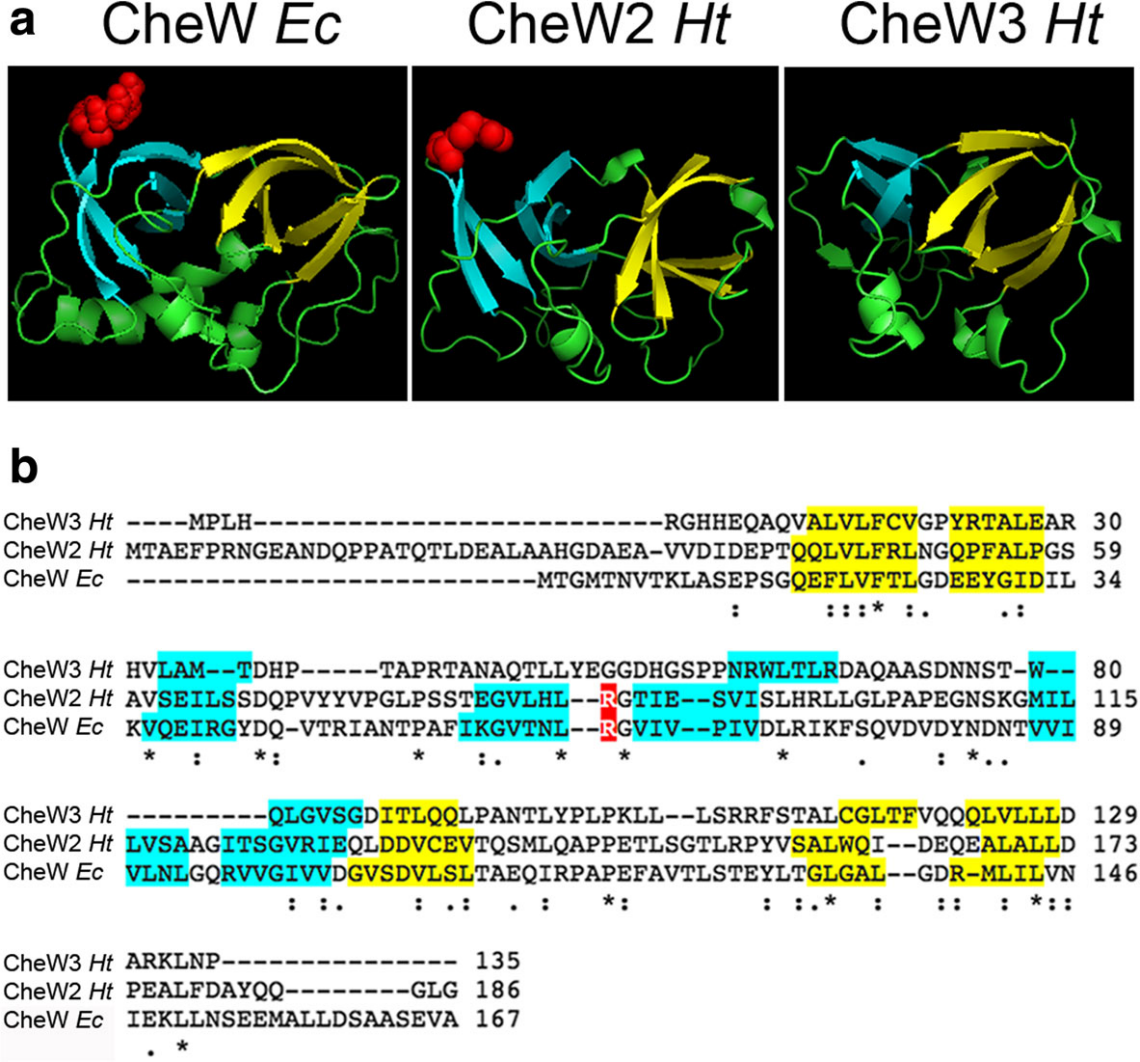
Predicted structure of CheW2 and CheW3 from *H. titanicae* KHS3. **a** Comparison between the template and models. Structure of the template CheW from *E. coli* (PDB accession code 2HO9, left) and the models obtained with SWISSMODEL for CheW2 *Ht* (middle) and CheW3 *Ht* (right). The β-strands that form the two β-barrels are colored yellow (subdomain 1) or cyan (subdomain 2). The conserved residue R62 in subdomain 2 of CheW *Ec* is shown as red spheres. **b** Clustal Omega alignment of CheW *Ec*, CheW2 *Ht* and CheW3 *Ht*. β-strands are color-coded as in A. Notice that subdomain 2 is incomplete in CheW3 *Ht*.

Whereas the CheW protein that is coded in chemosensory cluster 1 shows high identity compared with its *E.coli* homolog (almost 68%, Additional file 3: Table S2), chemosensory cluster 2 contains a gene that codes for a cheW with significantly lower identity percentage (19%, Additional file 3: Table S2), but shows considerable structural homology (Fig. 5). Contiguous to it lies an additional gene that codes for a hypothetical protein that does not show sequence homology with proteins of known function. However, both Phyre2 and SWISS-MODEL software modeled it as an aberrant CheW protein, hence named CheW3 (Fig. 5). The modeled protein shows a structure that clearly resembles CheW but lacks protein regions that had been described as essential for its coupling function. CheW consists of two β-barrels that sandwich a hydrophobic core [44, 45]. Whereas the first subdomain has been shown to connect core complexes in the chemoreceptor array [46], the second subdomain interacts with the kinase CheA to form the active core complexes, and is essential to mediate kinase control [47, 48]. In CheW3, however, some β-strands are missing (Fig. 5a, b) so that the second subdomain is not complete. This suggests that CheW3 could be a non-functional protein or it might play a novel role.

The cluster *wsp* from *P. aeruginosa* also codes for two CheW proteins (WspB and WspD), but in this case none of them deviates from the conserved two-barrel characteristic structure (not shown).

#### Diguanylate cyclase (DGC)

**Fig. 6:**
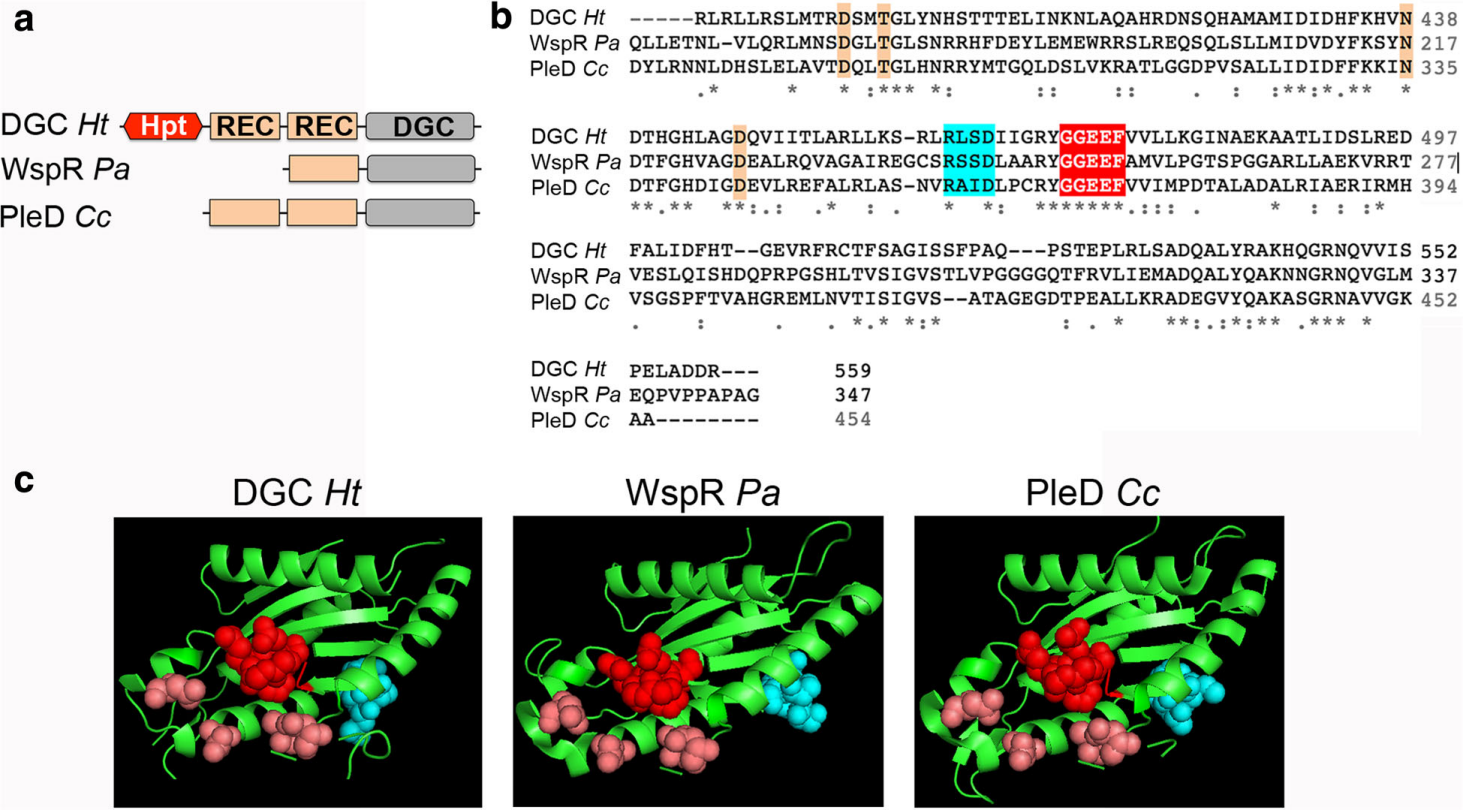
Predicted structure of the diguanylate cyclase (chemosensory cluster 2) from *H. titanicae*. **a** Schematic representation of cluster 2 diguanylate cyclase from *H. titanicae* (DGC *Ht*), WspR from *P. aeruginosa* (WspR *Pa*) and PleD from *C. crescentus* (PleD *Cc*). A red hexagonal shape indicates the histidine phosphotransfer domain (Hpt; receiver domains (REC) are indicated by light orange rectangles, and diguanylate cyclase catalytic domains (DGC) by gray rectangles. **b** Clustal Omega alignment of catalytic DGC domains from DGC *Ht*, WspR *Pa* and PleD *Cc*. Active site residues GGEEF are highlighted red, I-site residues RxxD are highlighted cyan, and other conserved residues that have been shown to be involved in activity are highlighted orange (**c**) Modeled structure for the catalytic domain from DGC *Ht* compared with the corresponding crystal structures from WspR *Pa* (PDB accession code 3BRE) and PleD *Cc* (PDB accession code). Residues shown as spheres are color-coded as in B

The predicted DGC coded in cluster 2 contains a phosphotransfer Hpt domain followed by two receiver domains, and the second one is connected to the catalytic domain through a short linker (Fig. 6a); all three domains putatively involved in phosphorelay signaling do contain the potentially phosphorylatable residues, based on alignments with the corresponding domains of known signaling proteins (Additional file 5: Figure S3). This domain organization differs from WspR, the diguanylate cyclase from cluster *wsp*, which contains only one receiver domain and a catalytic domain that are connected via a long alpha-helix [49].

The presence of two contiguous receiver domains makes the DGC from cluster 2 very similar to PleD from *Caulobacter crescentus*, although the latter lacks the Hpt domain (Fig. 6a). The catalytic domain from cluster 2 DGC contains most of the residues that have been described as important for diguanylate cyclase enzymatic activity (Fig. 6b, c). The active site (A-site), with the sequence GG(D/E)EF, binds GTP through the first two glycine residues and the third residue, which is acidic, is indispensable for catalysis [50]. Many DGC proteins also have an I-site (Inhibitory site) formed by a four-residue sequence “RxxD” usually located five amino acids upstream from the A-site [51]. *H. titanicae* DGC also has an I-site (Fig. 6b), as well as some conserved residues in the N-terminal region of the catalytic domain like the “DxLT” motif, that is commonly found at the beginning of all GGDEF domains and seems to be involved in DGC activity [50] (Fig. 6b, c). DGC from *H. titanicae* cluster 2 seems to be specific for GTP, as it possesses the residues that have been described for nucleotide specificity (N335 and D344 in PleD, [50] (Fig. 6b, c and Additional file 5: Figure S3).

Based on the analysis of *H. titanicae* KHS3 chemosensory clusters, we speculate that the “canonical” cluster 1 is responsible for the general chemotactic behavior of *H. titanicae* KHS3 mediated by flagella, through the control of CheA1 by all the chemoreceptors encoded in the genome, with the exception of the one that belongs to cluster 2. As for chemosensory cluster 2, its output seems to be exclusively controlled by the chemoreceptor encoded within the cluster, resulting in the modulation of the activity of the diguanylate cylase. Whether the c-di-GMP levels affect the ability of *H. titanicae* cells to form biofilms or a different physiological process will remain as a question whose answer will require more experimental work.

### Phylogenetic distribution of chemosensory clusters 1 and 2

An analysis of all the representatives of the genus *Halomonas* whose genomic sequences are available in IMG database at the moment of publication (Additional file 1: Table S1) showed that only 17 out of 55 *Halomonas* strains contained the same two clusters of chemosensory genes described above for *H. titanicae* KHS3. Seven *Halomonas* strains lack any chemosensory gene, one strain (*Halomonas* sp. KO116) only has chemosensory cluster 2 and the remaining 30 strains only contained the canonical cluster 1. To shed light on the phylogenetic relationships between the species containing or lacking the chemosensory cluster 2, we built two different phylogenetic trees.

**Fig. 7:**
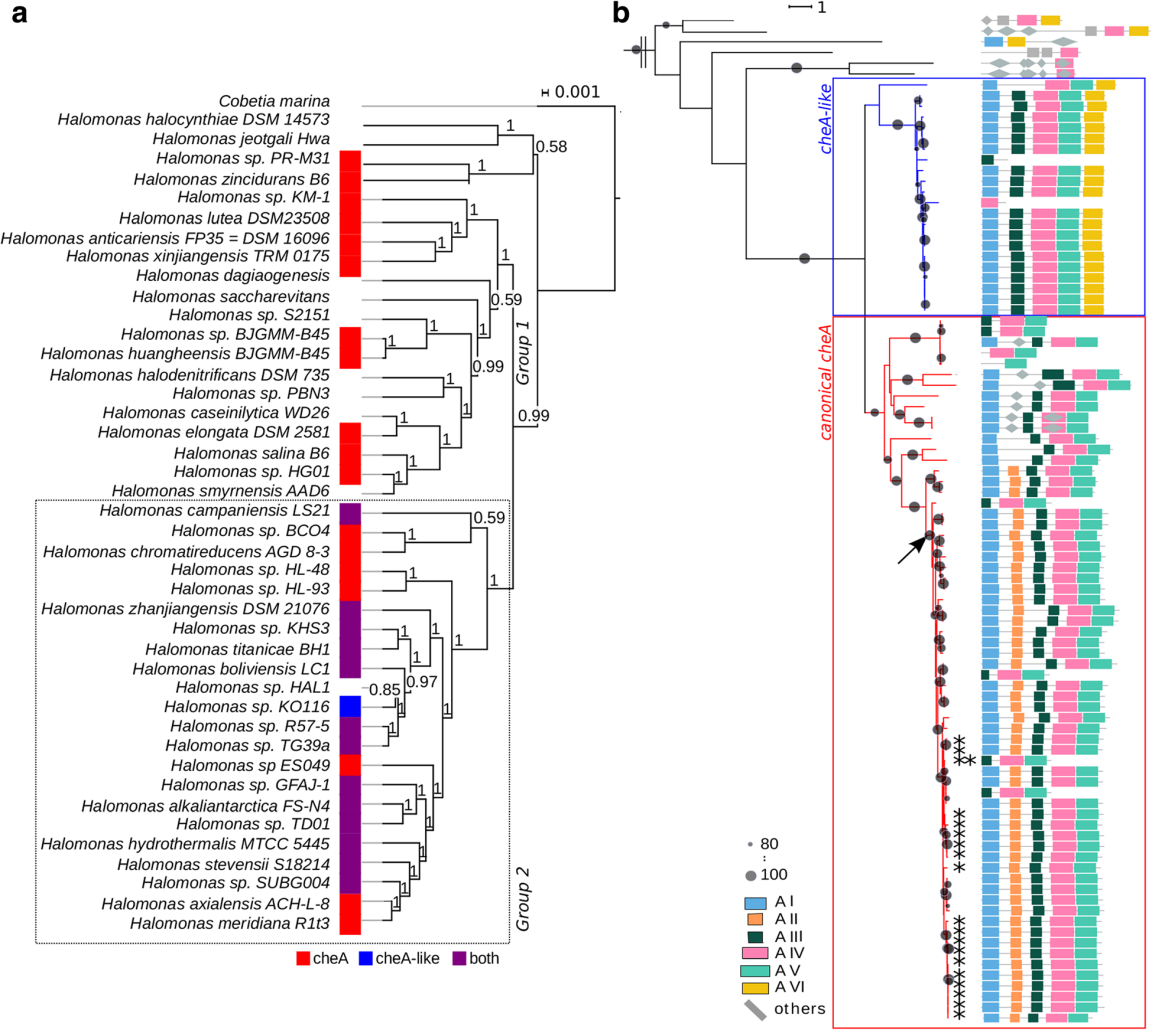
CheA and CheA-like protein phylogeny largely follows taxonomy-based topology. **a** 16S + 23S *Halomonas sp.* Phylogenetic Tree. 16S and 23S data were retrieved from the available *Halomonas sp.* genomes and aligned with SINA, specifying SSU or LSU, respectively. Both alignments were attached and phylogenetic analyses were performed with BEAST package. The colored squares indicate the presence of canonical cheA sequence only (red), cheA-like sequence only (blue) or both types (purple); no colored squares indicate lack of both cheAs in those genomes. This tree shows a main bifurcation that largely explains the canonical CheA / CheA-like clade separation (dot line square enclosing the group 2 where *Halomonas* strains with two clusters are included). **b** CheA protein phylogenetic analyses. In red, canonical CheA clade. In blue, CheA-like clade. Protein domains are depicted for each sequence. The black arrow indicates the root of the *Halomonas* sp. CheA clade. Asterisks indicate strains that have the two clusters. Big gray circles represent bootstrap support 100%; small gray circles represent bootstrap support 80%. The two lines at the start of the tree indicate where it was trimmed. The complete unrooted tree can be seen in Additional file 5: Figure S3

The first tree was built using the 16S and 23S ribosomal RNA genes from all the *Halomonas* species whose genomes are available (Fig. 7a). In this tree, all the species belong to one of two clearly distinct groups, in agreement with previous reports [51]. The type strain of the genus (*H. elongata*) is situated within Group 1, whereas *H. titanicae* KHS3 belongs to Group 2, the same as all the *Halomonas* strains containing two chemosensory clusters.

The second tree was built using CheA sequences from the *Halomonas* species, and also some CheA sequences from other genera (Fig. 7b and Additional file 6: Figure S4). All the CheA-like sequences, that is, the genes coding for proteins with a domain organization similar to CheA2, form a very well defined and separate group (blue box), indicating that the non-canonical CheAs are related to each other. The other group, containing the CheA genes from canonical chemosensory cluster 1 (red box), is divided into two branches that correlate quite well with the two branches present in the rRNA-gene tree. Most of the strains that share the branch with *H. titanicae* KHS3 do contain the chemosensory cluster 2, suggesting that it was already present in the common ancestor of this branch.

#### Homologs to chemosensory cluster 2 from *H. Titanicae* in other species of Proteobacteria

The singularity of the gene composition/order observed in cluster 2, as well as the uniqueness of the diguanylase cyclase coded within this cluster prompted us to investigate whether it was restricted to the subgroup of *Halomonas* genus mentioned above.

A HMMER search using DGC from cluster 2 as query against reference proteomes identified 83 DGC sequences that showed exactly the same domain organization as DGC from cluster 2. From the first 20 hits, 18 had their genome available enabling us to examine the genomic context of these DGCs. We found that 16/18 sequences belonged to gene clusters with exactly the same gene order/domain composition of the predicted genes in cluster 2 (Fig. 9).

Similarly, when the HMMER search was performed using CheA2 as query, the same 16 organisms with cluster 2-like sequences were found among the first 25 hits, together with sequences corresponding to similar clusters with some alteration in gene order or composition (Fig. 9).

**Fig. 8:**
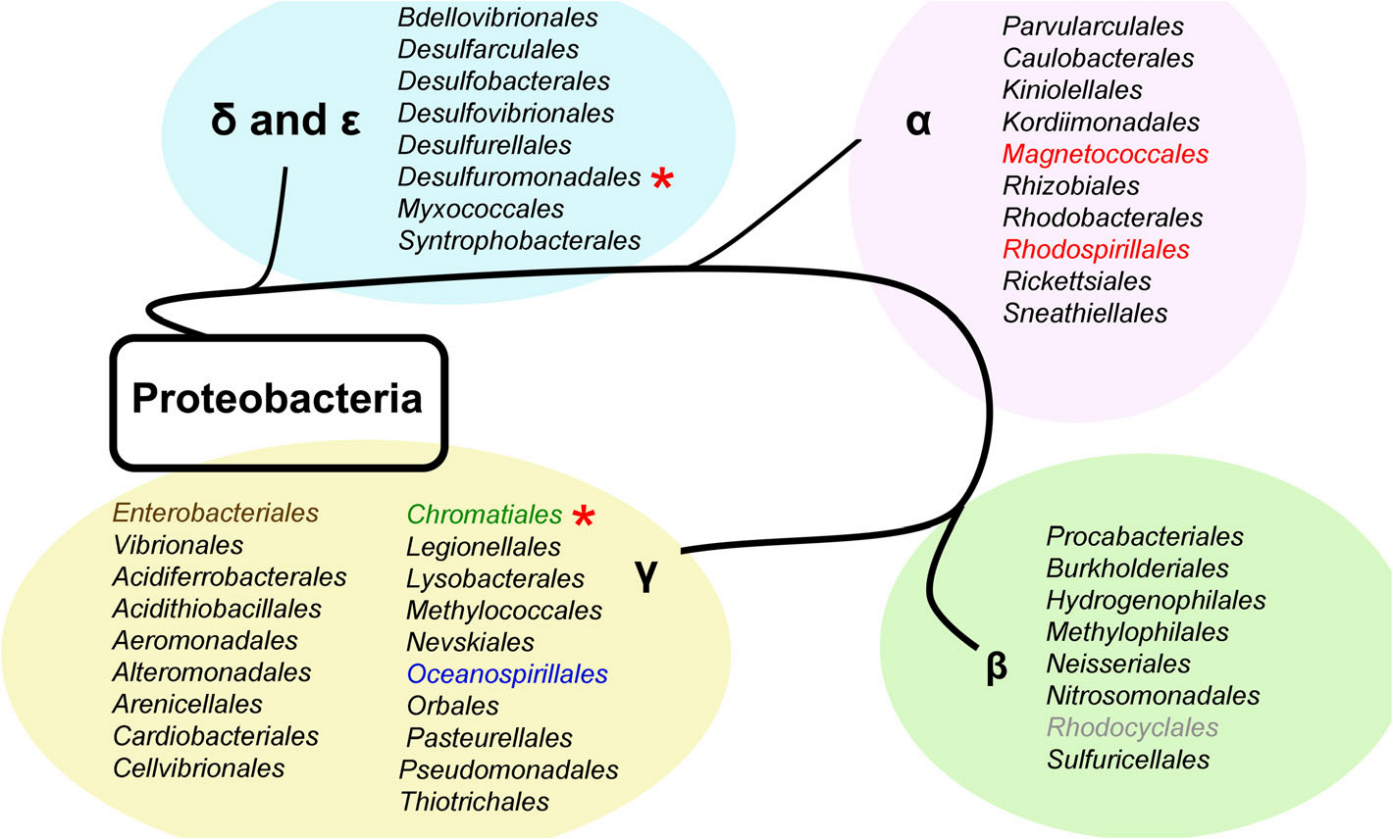
Schematic representation of the phylum *Proteobacteria*. Black lines indicate the sequence of appearance of *Proteobacteria* classes as described by Gupta et al. [56]: δ and ε-*Proteobacteria* (eight orders, light blue), α-*Proteobacteria* (ten orders, light pink), β-*Proteobacteria* (eight orders, light green) and γ-*Proteobacteria* (nineteen orders, light yellow). Within each class, orders that contain species that carry cluster 2-like sequences are written in color (color-code as in Fig. 9). Asterisks indicate orders where we identified gene clusters that resemble cluster 2 with some alteration in gene order/composition (see text)

**Fig. 9:**
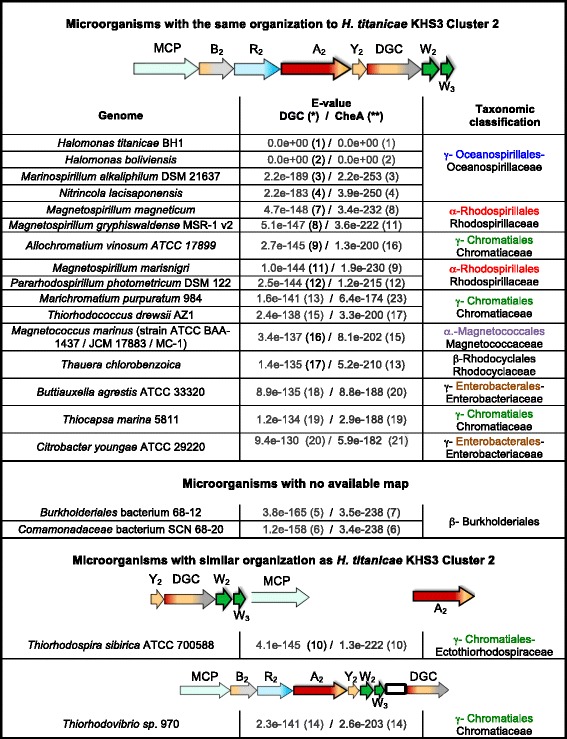
Chemosensory clusters similar to *H. titanicae* cluster 2 in other groups of *Proteobacteria.* (*) The number between parentheses indicates the order number for each protein in the list of the first 20 hits of a Phmmer search using *H.titanicae* Cluster 2 DGC as query. (**) The number between parentheses indicates the order number for each protein in the list of hits of a Phmmer search using *H.titanicae* CheA2 as query

Remarkably, clusters with this organization are not restricted to the *Halomonas* genus (Order *Oceanospirillales,* Family *Halomonadaceae*). Cluster 2-like sequences are found in other families of the same order (Order *Oceanospirillales,* Family *Oceanospirillaceae*) as well as in different orders of Gammaproteobacteria (Order *Chromatiales* and Order *Enterobacteriales*) and even in some orders belonging to *Beta* (Order *Rhodocyclales*) and *Alphaproteobacteria* Classes (Order *Rhodospirillales* and Order *Magnetococcales*) (Fig. 8). In most cases both gene order and domain composition of the predicted proteins is strictly maintained and identical to the *H. titanicae* KHS3 cluster 2 (Fig. 9), suggesting that all of them share a common origin and/or common functional features.

We speculate that the most likely explanation for the presence of chemosensory cluster 2-like sequences in different phylogenetic groups must include some lateral gene transfer events, followed by vertical transmission and diversification within the groups. Identity matrices made with the genes coding for CheA, DGC and CheR from the 16 most similar clusters and *H. titanicae* KHS3 show that the identity values are higher within the same order (Additional file 7: Table S3).

CheA and DGC proteins with the domain composition showed by cluster 2 representatives but with lower E-values in our HMMER searches were found within clusters lacking some components or showing some alteration in gene order (not shown). Notably, most of the alterations in gene order were of the circular permutation type, still suggesting a common origin. Such incomplete or altered “cluster 2-like” sequences were found in yet another branch of *Proteobacteria*, i.e. *Deltaproteobacteria*, with many variations among species belonging to *Geobacteraceae* family, Order *Desulfuromonadales* (see Fig. 8).

## Discussion

The hydrocarbon-degrading strain isolated from Mar del Plata harbor was identified as *Halomonas titanicae* KHS3. The strain that defined the species, *Halomonas titanicae* BH1, was isolated from rusticles (formations made of rust, that occur underwater when wrought iron oxidizes) from the RSM *Titanic* wreck site [29]. Several genes related to metal corrosion were identified in its genome [52], explaining its presence on metal surfaces from ships, and perhaps its occurrence in contaminated harbors around the world. Analysis of publicly available sequences of *Halomonas* strains during this work, allowed the identification of two additional *Halomonas titanicae* strains, i.e. *Halomonas* sp. 19A GOM-1509 m, isolated from the Deepwater Horizon oil spill site that occurred in 2010 in the Gulf of Mexico, and *Halomonas* sp. A3H3, isolated from another contaminated site at a harbor in the south of France [53]. Isolation from hydrocarbon-contaminated sites suggests that they also have degradation capabilities.

Chemotaxis plays a key role in many processes. It has been shown that chemotactic responses to xenobiotic compounds represent an advantage for biodegradation [5, 6]. *H. titanicae* KHS3 displays chemotactic behavior to several compounds, including polyaromatic hydrocarbons and intermediate metabolites (Fig. 1). The ability to sense phenanthrene and use it as sole carbon source is also present in *H. titanicae* BH1, indicating that it might be spread among all the strains of *H. titanicae*.

We found a “canonical” cluster (cluster 1) of chemotaxis genes in 48 out of 56 of the *Halomonas* strains with available genomes including *H. titanicae* KHS3. Both the gene order (Fig. 2b) and sequence conservation (Additional file 1: Table S1) strongly suggest that cluster 1 is responsible for the general chemotactic behavior. The effectors that feed into this pathway depend on the number and variety of chemoreceptors that associate with and control the activity of the CheA1 kinase. In *H. titanicae* KHS3, presumably 24 out of the 25 MCPs are involved in the control of the general chemotactic behavior. All these 24 MCPs belong to the same length class (36H), indicating that they are capable to assemble together in the same chemoreceptor array, and 11 of them possess the C-terminal pentapeptide that is presumably recognized for the methyltransferase CheR1 to drive adaptation responses (Fig. 3).

A second chemosensory system (cluster 2) resembles the *P. aeruginosa* Wsp transduction pathway, which is involved in biofilm formation [32]. Similarly to the Wsp pathway, cluster 2 contains genes that code for a chemoreceptor of the 40H class, a histidine kinase that contains a receiver domain at its C-terminus (CheA2), a methyltransferase with a tetratricopeptide repeat domain at its C-terminus (CheR2), and a diguanylate cyclase with receiver domains preceding the catalytic domain (DGC *Ht* in this work), suggesting that the pathway proceeds from an unknown stimulus to the control of c-di-GMP levels. However, cluster 2 displays a clearly different gene organization, and includes additional genes and domains. The actual function of cluster 2 in *Halomonas* physiology can only be speculative until genetic, biochemical and behavioral experiments are conducted. However, the gene composition of cluster 2 allows its classification into the gene clusters that are involved in the control of alternative cellular functions other than flagellar control [7].

Even though TPR domains have been involved in protein-protein interactions [54], no specific partners for methyltransferase-TPR from chemosensory pathways have been found up to date. Probably, as has been described for other CheR-TPR proteins [33, 43] the TPR domain from CheR2 from *H. titanicae* KHS3 somehow affects the methylation of the chemoreceptor coded in cluster 2.

The diguanylate cyclase that is coded in cluster 2 contains an N-terminal phosphotransfer domain (Hpt), followed by two receiver domains (Rec) and the catalytic domain. Alignments of the individual domains with known signaling proteins show the presence of the potentially phosphorylatable residues, suggesting that a phosphorelay cascade controls the catalytic activity. Up to our knowledge, there are no characterized diguanylate cyclases with this domain organization. Among the ones that have been characterized, the most similar enzyme is PleD from *C. crescentus*, which contains two receiver domains in tandem. This enzyme dimerizes upon phosphorylation of one of the receiver domains to become active [55]. Unlike PleD, DGC *Ht* contains potentially phosphorylatable residues in both receiver domains, as well as in the Hpt domain. Experimental work will be needed to determine whether such phosphorylation occurs, and its functional consequences.

The fact that the only chemoreceptor coded within cluster 2 belongs to a different length class than all the other chemoreceptors coded in the genome seems to indicate that the activity of this pathway is controlled exclusively by this receptor. It has been demonstrated that the difference in length between receptors of the 36H and the 40H class is enough to avoid the assembly of these two kinds of receptors into the same array [38], suggesting that cluster 2 proteins assemble and signal independently.

Chemosensory cluster 2 is present in *Halomonas* species belonging to group 2 and absent from species of group 1. This suggests either duplication from chemosensory cluster 1 in the group 2 ancestor, with a posterior functional diversification, or a horizontal transfer event into this ancestor. Chemosensory cluster 1 is present in two *Halomonas* species (*H. zincidurans* B6 and strain PR-M31) (Fig. 7a), which are outside our strict group 1 definition, and this could be explained by the proposed polyphyletic nature of this genus [51]. Notably, we identified gene clusters with a strict conservation of gene order and domain composition within other marine species belonging to different proteobacterial lineages (Fig. 8). The first hits of HMMER searches using either the diguanylate cyclase or the CheA2 from cluster 2 as queries, identified proteins belonging to gene clusters that share the organization of cluster 2. This fact suggests that some characteristics of these two proteins make them specific for this type of cluster. The conserved association between cluster 2 genes could derive from a common origin that might include lateral gene transfer events within the marine environment.

## Conclusions

The physiology of many *Halomonas* strains has been deeply analyzed in the last years due to the increasing use of microorganisms from this genus in biotechnological processes. This study is the first genomic investigation of chemosensory systems in *Halomonas* strains.

Two chemotaxis-related genes clusters were found in the genomic sequence of *H. titanicae* KHS3, a hydrocarbon-degrading microorganism isolated from contaminated seawater. The same two-chemosensory cluster-organization was found in several representatives from the Group 2 of *Halomonas* genus. It is worth noting that several strains that have been shown in this work to have the cluster 2 [(*H. campaniensis* and *H. boliviensis* (PHAs production), *H. alkaliantarctica* (EPS producer), *H. stevensii* (human pathogen)] are strains studied intensely due to its use in biotechnological processes. Information obtained in this work constitutes the first step to the study of chemotaxis and its implications in biotechnological applications of *Halomonas* strains.

## Additional files

Additional file 1: **Table S1.** Title: List of genomes of *Halomonas* species included in the phylogenetic analysis. Species shaded in gray were not included in the taxonomy phylogeny due to missing 16S and/or 23S data. Genomic sequences were obtained from NCBI, ENSEMBL BACTERIA, PATRIC. Gene predictions were obtained from NCBI or obtained using GenMark, IMG and RAST servers. Supplemental Citations: [57, 58]. *software tools:* GeneMark. [59]. (XLS 32 kb)

Additional file 2: **Figure S1.** Chemotactic behavior of *Halomonas titanicae* strains KHS3 and BH1. Minimal medium H1 soft-agar plates containing different carbon and energy sources (as indicated) were prepared as described in *Methods*. Strains in the plates: no-QT (a chemotaxis defective derivative of *H. titanicae* KHS3); BH1 (*H. titanicae* BH1); KHS3 (*H. titanicae* KHS3). Bacteria were inoculated in the center of the plate and incubated at 28-30 °C for 24-48 h. (TIF 5433 kb)

Additional file 3: **Table S2.** Identity values between proteins from *E. coli che* cluster and proteins from *H. titanicae* KHS3 clusters 1 and 2. Values were obtained from alignments made with Clustal Omega. (DOCX 14 kb)

Additional file 4: **Figure S2.** Clustal Omega alignment of complete CheR proteins. Protein sequences of *P. aeruginosa* CheR1, CheR2 and CheR3 (PA3348, PA0175 and PA0412 respectively), *S. typhimurium* CheR (P07801), *E. coli* CheR (P07364) and *H. titanicae* CheR1 and CheR2 (in bold letters, RO22_21465 and RO22_21165 respectively) were aligned with Clustal Omega. Residues that have been described as important for the interaction with the tryptophan residue of chemoreceptor C-terminal pentapeptide, that is the β-loop subdomain (residues GTGPH in *E. coli* and *S. typhimurium* cheRs) and residue R197, are fuchsia and light blue shaded, respectively. Residues that are highly conserved in the active site of chemoreceptor methyltransferases (R98, D154 and Y235 in CheR *St*) are red shaded. Alternate gray and yellow shading indicate the tetratricopeptide repeats at the C-terminus of CheR3 *Ht* and WspC *Pa*. Clustal Omega color-code for aminoacids: Red letters: small and hydrophobic residues; blue letters: acidic residues; magenta letters: basic residues; green letters: hydroxyl+ sulphydryl+amine+G; gray letters: unusual aminoacids. * (Asterisk) indicates positions which have a single, fully conserved residue;**:** (colon) indicates conservation between groups of strongly similar properties; **.** (Period) indicates conservation between groups of weakly similar properties. (PDF 73 kb)

Additional file 5: **Figure S3.** Clustal Omega alignment of complete diguanylate cyclase proteins. Sequences of DGC *Ht* (RO22_21180), WspR *Pa* (PA3702) and PleD *Cc* (CC_2462), were aligned using Clustal Omega. The histidine phosphotransfer domain from DGC *Ht* is highlithed yellow; the putative phosphorylatable histidine residue is indicated in red. Receiver domains are highlighted in light/dark gray shades*;* phosphorylatable aspartate residues from WspR *Pa* and PleD *Cc* are indicated in red letters, as the potentially phosphorylatable aspartate residues in both Rec domains from DGC *Ht*. Important residues for catalytic activity are highlighted as in Fig. 6. (PDF 47 kb)

Additional file 6: **Figure S4.** Unrooted CheA phylogenetic tree, with two black lines showing where it was trimmed. Red branches indicate canonical CheAs and blue branches are the CheA-like proteins. Gray circles represent bootstrap support > 80%. (PDF 31 kb)

Additional file 7: **Table S3.** Protein percent identity matrices between the 16 microorganisms where clusters identical to cluster 2 were found. **A)** Percent identity matrix for diguanylate cyclase proteins from cluster 2-like sequences; **B)** Percent identity matrix for CheA proteins from cluster 2-like sequences; **C)** Percent identity matrix for CheR proteins from cluster 2-like. In blue: γ- Oceanospirillales, in red: α-Rhodospirillales, in green: γ- Chromatiales, in brown: γ- Enterobacterales. (PDF 61 kb)

## Abbreviations

ANI: Average nucleotide identity
c-di-GMP: Cyclic diguanylate
DDH: DNA-DNA Hybridization
DGC: Diguanylate cyclase
GGDC: Genome-to-genome distance calculation
LBD: Ligand-binding domain
MCP: Methyl-accepting chemotaxis proteins

## References

[1] Argandoña M, Vargas C, Reina-Bueno M, Rodriguez-Moya J, Salvador M, Nieto JJ (2012). An extended suite of genetic tools for use in bacteria of the Halomonadaceae: an overview. Methods Mol Biol.

[2] Rivera-Terceros P, Tito-Claros E, Torrico S, Carballo S, Van-Thuoc D, Quillaguaman J (2015). Production of poly(3-hydroxybutyrate) by *Halomonas boliviensis* in an air-lift reactor. J Biol Res (Thessalon).

[3] Garcia MT, Ventosa A, Mellado E (2005). Catabolic versatility of aromatic compound-degrading halophilic bacteria. FEMS Microbiol Ecol.

[4] Fathepure BZ (2014). Recent studies in microbial degradation of petroleum hydrocarbons in hypersaline environments. Front Microbiol.

[5] Paul D, Singh R, Jain RK (2006). Chemotaxis of *Ralstonia* sp. SJ98 towards p-nitrophenol in soil. Environ Microbiol.

[6] Parales RE, Harwood CS (2002). Bacterial chemotaxis to pollutants and plant-derived aromatic molecules. Curr Opin Microbiol.

[7] Wuichet K, Zhulin IB (2010). Origins and diversification of a complex signal transduction system in prokaryotes. Sci Signal.

[8] Lacal J, Garcia-Fontana C, Munoz-Martinez F, Ramos JL, Krell T (2010). Sensing of environmental signals: classification of chemoreceptors according to the size of their ligand binding regions. Environ Microbiol.

[9] D'Ippolito S, De Castro RE, Herrera Seitz MK (2011). Chemotactic responses to gas oil of *Halomonas* spp. strains isolated from saline environments in Argentina. Rev Argent Microbiol.

[10] Gasperotti AF, Studdert CA, Revale S, Herrera Seitz MK (2015). Draft genome sequence of *Halomonas* sp. KHS3, a Polyaromatic hydrocarbon-chemotactic strain. Genome Announc.

[11] Goris J, Konstantinidis KT, Klappenbach JA, Coenye T, Vandamme P, Tiedje JM (2007). DNA-DNA hybridization values and their relationship to whole-genome sequence similarities. Int J Syst Evol Microbiol.

[12] Richter M, Roselló-Mora R, Glöckner FO, Peplies J (2016). JSpeciesWS: a web server for prokaryotic species circumscription based on pairwise genome comparison. Bioinformatics.

[13] Auch AF, von Jan M, Klenk HP, Goker M (2010). Digital DNA-DNA hybridization for microbial species delineation by means of genome-to-genome sequence comparison. Stand Genomic Sci.

[14] Aziz RK, Bartels D, Best AA, DeJongh M, Disz T, Edwards RA, Formsma K, Gerdes S, Glass EM, Kubal M, Meyer F, Olsen GJ, Olson R, Osterman AL, Overbeek RA, McNeil LK, Paarmann D, Paczian T, Parrello B, Pusch GD, Reich C, Stevens R, Vassieva O, Vonstein V, Wilke A, Zagnitko O (2008). The RAST server: rapid annotations using subsystems technology. BMC Genomics.

[15] Markowitz VM, Chen IM, Palaniappan K, Chu K, Szeto E, Grechkin Y, Ratner A, Jacob B, Huang J, Williams P, Huntemann M, Anderson I, Mavromatis K, Ivanova NN, Kyrpides NC (2012). IMG: the integrated microbial genomes database and comparative analysis system. Nucleic Acids Res.

[16] Jones P, Binns D, Chang HY, Fraser M, Li W, McAnulla C, McWilliam H, Maslen J, Mitchell A, Nuka G, Pesseat S, Quinn AF, Sangrador-Vegas A, Scheremetjew M, Yong SY, Lopez R, Hunter S (2014). InterProScan 5: genome-scale protein function classification. Bioinformatics.

[17] Kelley LA, Mezulis S, Yates CM, Wass MN, Sternberg MJ (2015). The Phyre2 web portal for protein modeling, prediction and analysis. Nat Protoc.

[18] Arnold K, Bordoli L, Kopp J, Schwede T (2006). The SWISS-MODEL workspace: a web-based environment for protein structure homology modelling. Bioinformatics.

[19] Zimmermann L, Stephens A, Nam SZ, Rau D, Kübler J, Lozajic M, Gabler F, Söding J, Lupas AN, Alva V (2017). A completely Reimplemented MPI bioinformatics toolkit with a new HHpred server at its Core. J Mol Biol.

[20] Solovyev V, Salamov A. Automatic annotation of microbial genomes and metagenomic sequences. In: Li RW, editor. Metagenomics and its applications in agriculture, biomedicine and environmental studies. New York: Nova Science Publishers; 2011. p. 61–78.

[21] Pruesse E, Peplies J, Glockner FO (2012). SINA: accurate high-throughput multiple sequence alignment of ribosomal RNA genes. Bioinformatics.

[22] Bouckaert R, Heled J, Kuhnert D, Vaughan T, Wu CH, Xie D, Suchard MA, Rambaut A, Drummond AJ (2014). BEAST 2: a software platform for Bayesian evolutionary analysis. PLoS Comput Biol.

[23] Eddy SR (2011). Accelerated profile HMM searches. PLoS Comput Biol.

[24] Katoh K, Standley DM (2013). MAFFT multiple sequence alignment software version 7: improvements in performance and usability. Mol Biol Evol.

[25] Guindon S, Dufayard JF, Lefort V, Anisimova M, Hordijk W, Gascuel O (2010). New algorithms and methods to estimate maximum-likelihood phylogenies: assessing the performance of PhyML 3.0. Syst Biol.

[26] Huson DH, Scornavacca C (2012). Dendroscope 3: an interactive tool for rooted phylogenetic trees and networks. Syst Biol.

[27] Letunic I, Bork P (2011). Interactive tree of life v2: online annotation and display of phylogenetic trees made easy. Nucleic Acids Res.

[28] Hazelbauer GL, Park C, Nowlin DM (1989). Adaptational “crosstalk” and the crucial role of methylation in chemotactic migration by *Escherichia coli*. Proc Natl Acad Sci U S A.

[29] Sanchez-Porro C, Kaur B, Mann H, Ventosa A (2010). *Halomonas titanicae* sp. nov., a halophilic bacterium isolated from the RMS titanic. Int J Syst Evol Microbiol.

[30] Helmann JD, Chamberlin (1987). DNA sequence analysis suggests that expression of flagellar and chemotaxis genes in *Escherichia coli* and *Salmonella typhimurium* is controlled by an alternative sigma factor. Proc Natl Acad Sci U S A.

[31] D'Argenio DA, Calfee MW, Rainey PB, Pesci EC (2002). Autolysis and autoaggregation in *Pseudomonas aeruginosa* colony morphology mutants. J Bacteriol.

[32] Hickman JW, Tifrea DF, Harwood CS (2005). A chemosensory system that regulates biofilm formation through modulation of cyclic diguanylate levels. Proc Natl Acad Sci U S A.

[33] O’Connor JR, Kuwada NJ, Huangyutitham V, Wiggins PA, Harwood CS (2012). Surface sensing and lateral subcellular localization of WspA, the receptor in a chemosensory-like system leading to c-di-GMP production. Mol Microbiol.

[34] Hulko M, Berndt F, Gruber M, Linder J, Truffault V, Schultz A, Martin J, Scultz JE, Lupas AN (2006). The HAMP domain structure implies helix rotation in transmembrane signaling. Cell.

[35] Alexander RP, Zhulin IB (2007). Evolutionary genomics reveals conserved structural determinants of signaling and adaptation in microbial chemoreceptors. Proc Natl Acad Sci U S A.

[36] Shu CJ, Ulrich LE, Zhulin IB (2003). The NIT domain: a predicted nitrate-responsive module in bacterial sensory receptors. Trends Biochem Sci.

[37] Shiomi D, Zhulin IB, Homma M, Kawagishi I (2002). Dual recognition of the bacterial chemoreceptor by chemotaxis-specific domains of the CheR methyl transferase. J Biol Chem.

[38] Herrera Seitz MK, Frank V, Massazza DA, Vaknin A, Studdert CA (2014). Bacterial chemoreceptors of different length classes signal independently. Mol Microbiol.

[39] Inclán YF, Laurent S, Zusman DR (2008). The receiver domain of FrzE, a CheA-CheY fusion protein, regulates the CheA histidine kinase activity and downstream signalling to the A- and S-motility systems of *Myxococcus xanthus*. Mol Microbiol.

[40] Djordjevic S, Stock AM (1997). Crystal structure of the chemotaxis receptor methyltransferase CheR suggests a conserved structural motif for binding S-adenosylmethionine. Structure.

[41] Garcia-Fontana C, Corral Lugo A, Krell T (2014). Specificity of the CheR2 methyltransferase in *Pseudomonas aeruginosa* is directed by a C-terminal pentapeptide in the McpB chemoreceptor. Sci Signal.

[42] Muñoz Martínez F, García-Fontana C, Rico-Jiménez M, Alfonso C, Krell T (2012). Genes encoding CheR-TPR fusion proteins are predominantly found in gene clusters encoding chemosensory pathways with alternative cellular functions. PLoS One.

[43] Scott AE, Simon E, Park SK, Andrews P, Zusman DR (2008). Site-specific receptor methylation of FrzCD in *Myxococcus xanthus* is controlled by a tetra-trico peptide repeat (TPR) containing regulatory domain of the FrzF methyltransferase. Mol Microbiol.

[44] Griswold IJ, Zhou H, Matison M, Swanson RV, McIntosh LP, Simon MI, Dahlquist FW (2002). The solution structure and interactions of CheW from *Thermotoga maritima*. Nat Struct Biol.

[45] Li Y, Hu Y, Fu W, Xia B, Jin C (2007). Solution structure of the bacterial chemotaxis adaptor protein CheW from *Escherichia coli*. Biochem Biophys Res Commun.

[46] Piñas GE, Frank V, Vaknin A, Parkinson JS (2016). The source of high signal cooperativity in bacterial chemosensory arrays. Proc Natl Acad Sci U S A.

[47] Liu J, Hu B, Morado DR, Jani S, Manson MD, Margolin W (2012). Molecular architecture of chemoreceptor arrays revealed by cryoelectron tomography of *Escherichia coli* minicells. Proc Natl Acad Sci U S A.

[48] Briegel A, Li X, Bilwes AM, Hughes KT, Jensen GJ, Crane BR (2012). Bacterial chemoreceptor arrays are hexagonally packed trimers of receptor dimers networked by rings of kinase and coupling proteins. Proc Natl Acad Sci U S A.

[49] De N, Navarro MVAS, Raghavan RV, Sondermann H (2009). Determinants for the activation and autoinhibition or the diguanylate cyclase response regulator WspR. J Mol Biol.

[50] Schirmer T (2016). C-di-GMP synthesis: structural aspects of evolution, catalysis and regulation. J Mol Biol.

[51] de la Haba RR, Arahal DR, Marquez MC, Ventosa A (2010). Phylogenetic relationships within the family Halomonadaceae based on comparative 23S and 16S rRNA gene sequence analysis. Int J Syst Evol Microbiol.

[52] Sánchez Porro C, de la Haba RR, Cruz-Hernández N, González JM, Reyes-Guirao C, Navarro-Sampedro L, Carballo M, Ventosa A (2013). Draft genome of the marine Gammaproteobacterium *Halomonas titanicae*. Genome Announc.

[53] Koechler S, Plewniak F, Barbe V, Battaglia-Brunet F, Jost B, Joulian C, Philipps M, Vicaire S, Vincent S, Ye T, Bertin PN (2013). Genome sequence of *Halomonas* sp. strain A3H3, isolated from arsenic-rich marine sediments. Genome Announc.

[54] Cerveny L, Straskova A, Dankova V, Hartlova A, Ceckova M, Staud F, Stulik (2013). Tetratricopeptide repeat motifs in the world of bacterial pathogens: role in virulence mechanisms. J Infect Immun.

[55] Paul R, Abel S, Wassmann P, Beck A, Heerklotz H, Jenal U (2007). Activation of the diguanylate cyclase PleD by phosphorylation-mediated dimerization. J Biol Chem.

[56] Gupta RS (2016). The phylogeny of proteobacteria: relationships to other eubacterial phyla and eukaryotes. FEMS Microbiol Rev.

[57] Kersey PJ, et al. Ensembl Genomes 2016: more genomes, more complexity. Nucleic Acids Res. 2015:doi. 10.1093/nar/gkv1209. 10.1093/nar/gkv1209 PMC4702859 26578574

[58] Wattam AR (2014). PATRIC, the bacterial bioinformatics database and analysis resource. Nucl Acids Res.

[59] Besemer J, Borodovsky M (2005). GeneMark: web software for gene finding in prokaryotes, eukaryotes and viruses. Nucleic Acids Res.

