# Correction to: Identification of two different chemosensory pathways in representatives of the genus *Halomonas*

**DOI:** 10.1186/s12864-018-4811-x

**Published:** 2018-06-07

**Authors:** Ana Florencia Gasperotti, María Victoria Revuelta, Claudia Alicia Studdert, María Karina Herrera Seitz

**Affiliations:** 1 Instituto de Investigaciones Biológicas, CONICET - Universidad Nacional de Mar del Plata, Mar del Plata, Argentina; 2 Department of Medicine, Hematology and Oncology Division, Weill Cornell Medicine, New York, NY 10065 USA; 3 Instituto de Agrobiotecnología del Litoral, CONICET - Universidad Nacional del Litoral, Santa Fe, Argentina

## Correction

Following the publication of this article [1], the authors noticed that Fig. 3 was missing. In that figure, one of the numbers corresponding to the *Halomonas* chemoreceptors was missing: namely, chemoreceptor 07070. The correct version of Fig. 3 has been included in this Correction.

**Fig. 3:**
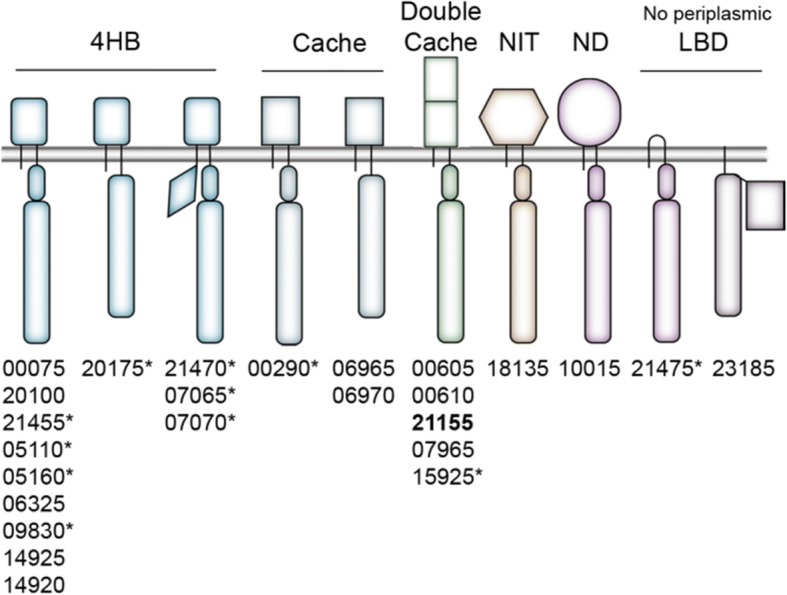
Schematic representation of chemoreceptors encoded in the genome of H. titanicae KHS3. MCPs are grouped according to the predicted structure of the periplasmic LBD: 4-helix bundle (4HB, rectangle with curved edges), Cache (rectangle), double Cache (double rectangle), nitrate-nitrite sensing fold (NIT, hexagon), not determined (ND, circle) and those with no periplasmic LBD. The gray horizontal bar represents the cytoplasmic membrane. MCP cytoplasmic subdomains are represented by a long rectangle (conserved cytoplasmic domain or signaling domain), an oval representing the HAMP domain, a diamond shape representing PAS domain. The rectangle at the C-terminus of RO22_23185 represents a Cache domain. The corresponding MCP ID numbers are listed below each kind of MCP (all ID numbers should be preceded by “RO22_” following the IMG gene annotation). Asterisks indicate the presence of C-terminal pentapeptide for interaction with CheR. All MCPs belong to the 36H family except one, which belongs to the 40H family (shown in bold)
