# Supplementary figures and images for: Identification of two different chemosensory pathways in representatives of the genus Halomonas
Source: BMC Genomics. 2018 Apr 18;19:266. doi: 10.1186/s12864-018-4655-4 (PMC5907407; doi:10.1186/s12864-018-4655-4)

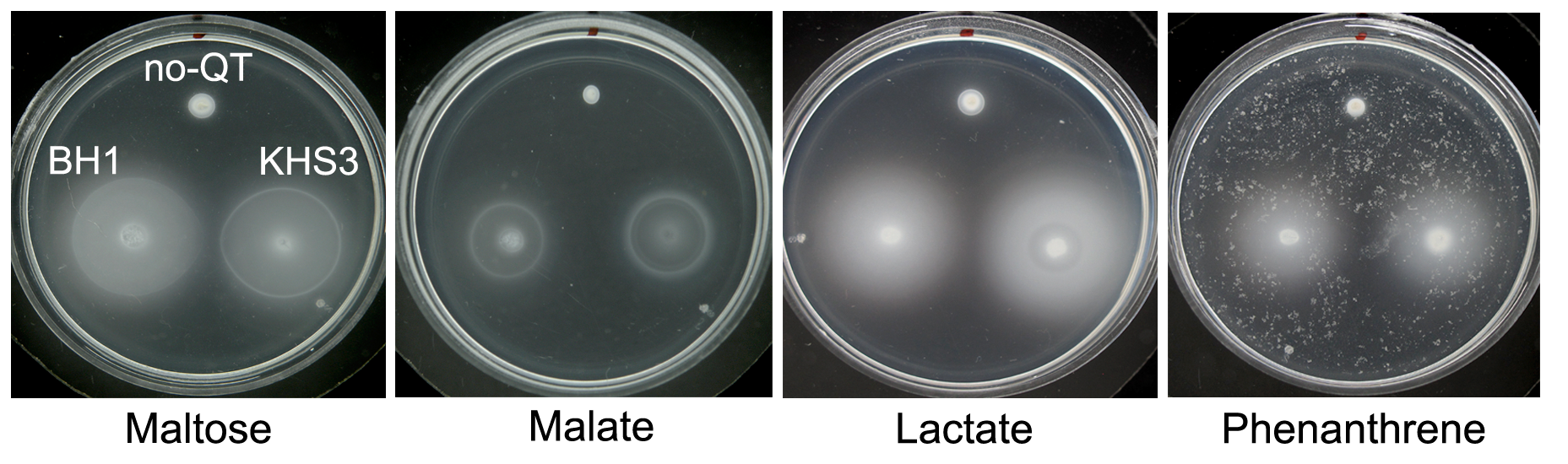

Supplement: Supplementary file 2 — Figure S1. Chemotactic behavior of Halomonas titanicae strains KHS3 and BH1. Minimal medium H1 soft-agar plates containing different carbon and energy sources (as indicated) were prepared as described in Methods. Strains in the plates: no-QT (a chemotaxis defective derivative of H. titanicae KHS3); BH1 (H. titanicae BH1); KHS3 (H. titanicae KHS3). Bacteria were inoculated in the center of the plate and incubated at 28-30 °C for 24-48 h. (TIF 5433 kb) [file 12864_2018_4655_MOESM2_ESM.tif]

Tree scale: 1

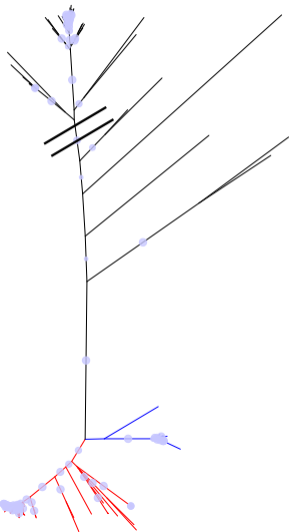

Supplement: Supplementary file 6 — Figure S4. Unrooted CheA phylogenetic tree, with two black lines showing where it was trimmed. Red branches indicate canonical CheAs and blue branches are the CheA-like proteins. Gray circles represent bootstrap support > 80%. (PDF 31 kb) [file 12864_2018_4655_MOESM6_ESM.pdf]
