# Supplementary material for: Identification of two different chemosensory pathways in representatives of the genus Halomonas
Source: BMC Genomics. 2018 Apr 18;19:266. doi: 10.1186/s12864-018-4655-4 (PMC5907407; doi:10.1186/s12864-018-4655-4)
Supplement: Supplementary file 3 — Table S2. Identity values between proteins from E. coli che cluster and proteins from H. titanicae KHS3 clusters 1 and 2. Values were obtained from alignments made with Clustal Omega. (DOCX 14 kb) [file 12864_2018_4655_MOESM3_ESM.docx]

|  | Protein | Identity (%) |
| --- | --- | --- |
| Cluster 1 | CheA1 | 58.6 |
|  | CheW1 | 67.9 |
|  | CheY1 | 82.2 |
|  | CheR1 | 56.3 |
|  | CheB1 | 69.0 |
|  | CheZ1 | 58.4 |
| Cluster 2 | CheA2 | 31.7 |
|  | CheW2 | 19.6 |
|  | CheW3 | 17.9 |
|  | CheY2 | 24.1 |
|  | CheR2 | 32.7 |
|  | CheB2 | 35.1 |
