# Supplementary material for: Identification of two different chemosensory pathways in representatives of the genus Halomonas
Source: BMC Genomics. 2018 Apr 18;19:266. doi: 10.1186/s12864-018-4655-4 (PMC5907407; doi:10.1186/s12864-018-4655-4)
Supplement: Supplementary file 4 — Figure S2. Clustal Omega alignment of complete CheR proteins. Protein sequences of P. aeruginosa CheR1, CheR2 and CheR3 (PA3348, PA0175 and PA0412 respectively), S. typhimurium CheR (P07801), E. coli CheR (P07364) and H. titanicae CheR1 and CheR2 (in bold letters, RO22_21465 and RO22_21165 respectively) were aligned with Clustal Omega. Residues that have been described as important for the interaction with the tryptophan residue of chemoreceptor C-terminal pentapeptide, that is the β-loop subdomain (residues GTGPH in E. coli and S. typhimurium cheRs) and residue R197, are fuchsia and light blue shaded, respectively. Residues that are highly conserved in the active site of chemoreceptor methyltransferases (R98, D154 and Y235 in CheR St) are red shaded. Alternate gray and yellow shading indicate the tetratricopeptide repeats at the C-terminus of CheR3 Ht and WspC Pa. Clustal Omega color-code for aminoacids: Red letters: small and hydrophobic residues; blue letters: acidic residues; magenta letters: basic residues; green letters: hydroxyl+ sulphydryl+amine+G; gray letters: unusual aminoacids. * (Asterisk) indicates positions which have a single, fully conserved residue;: (colon) indicates conservation between groups of strongly similar properties; . (Period) indicates conservation between groups of weakly similar properties. (PDF 73 kb) [file 12864_2018_4655_MOESM4_ESM.pdf]

```
CheR2 Ht   ---------------------------MSAFAPFKALVHQRCGLHLDGLAEARLFRAVAS   33
CheR3 Pa   -------MQANGVWSLQ----PLADMSAAEFRDWQVLLENRTGVVLNEQRRTFLQASLTA   49
WspC Pa    -----------------------------MNDRFERLLKSRIGLDASSVGSAVIERAVRQ   31
CheR1 Pa   -----------------------MSAANADFELFRVFLEKTCGIVLGSNKQYLVSSRLNK   37
CheR2 Pa   ----MPTSTP-----SPVFGNQEFHYTREDFQQVRERLYRLTGISLAESKAQLVYSRLSR   51
CheR1 Ht   MTEQRERVVDAGQWTSSSQIERDLVLTDADFTRIRELIYQRAGIVLAEHKREMVYSRLAK   60
CheR Ec    ----MTSSLPCGQTSLLLQMTERLALSDAHFRRISQLIYQRAGIVLADHKRDMVYNRLVR   56
CheR St    ----MTSSLPSGQTSVLLQMTQRLALSDAHFRRICQLIYQRAGIVLADHKRDMVYNRLVR   56
                                                :    *:         :   :

CheR2 Ht   LQASTGLTDITQLLKQLSCDT---ALFDQFVSQLTVNETYFFREPDALDWLVNTYLPQRL   90
CheR3 Pa   RMRELGIGDYHSYYRQVTDGPRGAVEWATLLDRLTVQETRFFRHPPSFELLERYLGERLR   109
WspC Pa    RMSGLALHDEDEYWMRLNGSP---GEVQALIEAVVVPETWFFRYPESFTTLARLAFERLP   88
CheR1 Pa   LMEQQGIKSLGELVQRIQTQ-RG-GLREMVVDAMTTNETLWFRDTYPFEVLKQRVLPELI   95
CheR2 Pa   RLRLLRLGSFAEYFTHLDREP---GEQQLFVNALTTNLTAFFRERHHFPLLADLARRQLQ   108
CheR1 Ht   RLRHHGMTRFTDYLVRLERQPEA-KEWEAFTNALTTNLTAFFREAHHFPLLAEHIKN---   116
CheR Ec    RLRSLGLTDFGHYLNLLESNQHS-GEWQAFINSLTTNLTAFFREAHHFPLLADHARR---   112
CheR St    RLRALGLDDFGRYLSMLEANQNS-AEWQAFINALTTNLTAFFREAHHFPILAEHARR---   112
                 :         :            . . :..  * :**    :  *

CheR2 Ht   -TTEQPPLSIFSAGCSSGEEPYSVAMMLFER---FGERAKALFTLTGGDLDHQVLAKARQ   146
CheR3 Pa   REGMPRPWALWSVGCSSGEEPYSLAMCAAQVLR--GQEREDFFGVTGTDISLHALQRARQ   167
WspC Pa    SLGGGRALRILSLPCSTGEEPYSIVMALLD-----AGLSEYLFEVDALDVSARVIERASL   143
CheR1 Pa   KANGGQRLRIWSAACSSGQEPYSLSMAIDEFEKTNLGQLKAGVQIVATDLSGSMLTAAKA   155
CheR2 Pa   ---RHRPLRIWSAAASTGEEPYSIAITLVE----ALGSFDPPVKIVASDIDTGVLDCARQ   161
CheR1 Ht   ---KQDPVTIWCSAASTGEEPYSLAMTLLE----TLGAKASQAKVIATDIDTDALARARA   169
CheR Ec    ---RSGEYRVWSAAASTGEEPYSIAMTLAD----TLGTAPGRWKVFASDIDTEVLEKARS   165
CheR St    ---RHGEYRVWSAAASTGEEPYSIAITLAD----ALGMAPGRWKVFASDIDTEVLEKARS   165
                    : .  .*:*:****: :   :              : . *:.   :  *

CheR2 Ht   AVYGGMA-FRALSPAFKKRYFS----PHKGRYKLHEPLRQWVTFRPFNLLNANEDSPVGP   201
CheR3 Pa   ANYPARK-LEQLEAGLVERYCERQ---ADGSFSVKTILTERVCCARLNVLDLAK-APWSG   222
WspC Pa    GVYGRNS-FRGDELGFRDRHFS----EVAEGYQLAEQVRRKVRFRCGNLLDPGLLAGEAP   198
CheR1 Pa   GEYDTLAMGRGLSPERLQRYFDAK---GPGRWAVKPAIRSRVEFRALNLLD-SY-ASLGK   210
CheR2 Pa   GVYPLER-LEQMPAPLKKRFFLRGTGPNAGKARVVEELRQLVEFRQINLLEADW-SIAGE   219
CheR1 Ht   GVYPLEQ-VRKIDEARVKRFFQKGTGNHAGFARIRPEVSALVEFLPLNLLAPQW-PIKGP   227
CheR Ec    GIYRHEE-LKNLTPQQLQRYFMRGTGPHEGLVRVRQELANYVDFAPLNLLAKQY-TVPGP   223
CheR St    GIYRLSE-LKTLSPQQLQRYFMRGTGPHEGLVRVRQELANYVEFSSVNLLEKQY-NVPGP   223
           . *      .       .*.             :   :   *     *:*        .

CheR2 Ht   FDVILFRNVSIYFDQQTRRHIHQQLSQLLAPNGILLCGVTETLGNDLGVFELTEAQGVFY   261
CheR3 Pa   MDVIFCQNLLIYFRRWRRREILNRLAERLAPGGLLVIGVGEVVDWSHPELEPVADERVLA   282
WspC Pa    YDFVFCRNLLIYFDRPTQSEVVEVLKRLLRSDGAMFIGPAEASLLSQHGMQPIGVPLSFV   258
CheR1 Pa   FDMVFCRNVLIYFSAEVKRDILLRIHGTLKPGGYLFLGASEALNNLPDHYQMVQCSPGII   270
CheR2 Pa   LDAIFCRNVMIYFDKPTQTRLLERMVALLRPEGLFFAGHSENFVHASHLVRSVGQTVYSP   279
CheR1 Ht   FDAVFCRNIMIYFDKDTQSKILKRFAPLMKPDGLLFAGHSENFSYISDAFKLRGQTVYNL   287
CheR Ec    FDAIFCRNVMIYFDQTTQQEILRRFVPLLKPDGLLFAGHSENFSHLERRFTLRGQTVYAL   283
CheR St    FDAIFCRNVMIYFDKTTQEDILRRFVPLLKPDGLLFAGHSENFSNLVREFSLRGQTVYAL   283
            * :: :*: ***    :  :   :   :   * :. *  *

CheR2 Ht   FRHAEQPAIVPETPLQPSLLAMDNMAEDSITEDSLSVAESANKQLALDNCTDVAPQAPRQ   321
CheR3 Pa   FTRKGYSGT---------------------------------------------------   291
WspC Pa    FRRTSEAPRGA----RPKAV----------SDGARPVVAAAVERASIR-PSPPPPAKPRQ   303
CheR1 Pa   YRAK--------------------------------------------------------   274
CheR2 Pa   A-----------------------------------------------------------   280
CheR1 Ht   AKK---------------------------------------------------------   290
CheR Ec    SKD---------------------------------------------------------   286
CheR St    SKDKA-------------------------------------------------------   288

CheR2 Ht   HATEKEPTD------SISHQLHTAHRLLNQNAFDDAATLLEALLEQQPWSIDALVLAGLV   375
CheR3 Pa   ------------------------------------------------------------   291
WspC Pa    RLSSLVPPASGQPLASPVGEFDEIARLADAGQHREARAACERQLAARGPSATVFYWLGLL   363
CheR1 Pa   ------------------------------------------------------------   274
CheR2 Pa   ------------------------------------------------------------   280
CheR1 Ht   ------------------------------------------------------------   290
CheR Ec    ------------------------------------------------------------   286
CheR St    ------------------------------------------------------------   288
```

```
CheR2 Ht      ARWQQRPQLAYEHFKRAIYVAPECWPAHFYLAELYR-QGELAD-KPLQRKQRYAAVVRLL   433
CheR3 Pa      ------------------------------------------------------------   291
WspC Pa       SDVAGQEQEAQDFYRKALYLEPQHAEALAHLAALLAARGDHAGARRLQQR--A-------   414
CheR1 Pa      ------------------------------------------------------------   274
CheR2 Pa      ------------------------------------------------------------   280
CheR1 Ht      ------------------------------------------------------------   290
CheR Ec       ------------------------------------------------------------   286
CheR St       ------------------------------------------------------------   288

CheR2 Ht      TTAPTSDGGLDTITPPLPPGDARFLAERYLDAVNLTLASTSTTQGVG   480
CheR3 Pa      -----------------------------------------------   291
WspC Pa       ARGVNKDG---------------------------------------   422
CheR1 Pa      -----------------------------------------------   274
CheR2 Pa      -----------------------------------------------   280
CheR1 Ht      -----------------------------------------------   290
CheR Ec       -----------------------------------------------   286
CheR St       -----------------------------------------------   288
```
