# Supplementary material for: Identification of two different chemosensory pathways in representatives of the genus Halomonas
Source: BMC Genomics. 2018 Apr 18;19:266. doi: 10.1186/s12864-018-4655-4 (PMC5907407; doi:10.1186/s12864-018-4655-4)
Supplement: Supplementary file 5 — Figure S3. Clustal Omega alignment of complete diguanylate cyclase proteins. Sequences of DGC Ht (RO22_21180), WspR Pa (PA3702) and PleD Cc (CC_2462), were aligned using Clustal Omega. The histidine phosphotransfer domain from DGC Ht is highlithed yellow; the putative phosphorylatable histidine residue is indicated in red. Receiver domains are highlighted in light/dark gray shades; phosphorylatable aspartate residues from WspR Pa and PleD Cc are indicated in red letters, as the potentially phosphorylatable aspartate residues in both Rec domains from DGC Ht. Important residues for catalytic activity are highlighted as in Fig. 6. (PDF 47 kb) [file 12864_2018_4655_MOESM5_ESM.pdf]

CLUSTAL O(1.2.4) multiple sequence alignment

```
DGC Ht          MSKPAPSLHDKLNQLRERFIEQLPSRLAQTAELWQQSRTTSEEQARLAPELHRFFHSLKG   60
WspR Pa         ------------------------------------------------------------   0
PleD Cc         ------------------------------------------------------------   0


DGC Ht          TGRSLGFERLAQLADQAQEALTTSPARADIDTFISQLLLLMGHEQQHLRSHQGQQQALAA   120
WspR Pa         -----------------------------------------MHNPHESKTDLG-------   12
PleD Cc         ------------------------------------------------------------   0


DGC Ht          VNSFELSSQVEPLRNKRQRLIYLCDDEPEQVDQLIHHLR-CFGHEVVQFIDTDTFFNAVL   179
WspR Pa         ----------APLD--GAVMVLLVDDQAMIGEAVRRSLASEAGIDFHFCSDPQQAVAVAN   60
PleD Cc         ----------------MSARILVVDDIEANVRLLEAKLT-AEYYEVSTAMDGPTALAMAA   43
                                    : : **       :   *      :.    *    .  .

DGC Ht          TRRPDAVIMDVQFPQGQTAGTETLTSLNKLTG----QPLPAIVLSAHSDFHSRLSAVRAG   235
WspR Pa         QIKPTVILQDLVMP-----GVDGLTLLAAYRGNPATRDIPIIVLSTKEEPTVKSAAFAAG   115
PleD Cc         RDLPDIILLDVMMP-----GMDGFTVCRKLKDDPTTRHIPVVLITALDGRGDRIQGLESG   98
                   *  :: *: :*     * : :*      .    : :* ::::: .    :  .. :*

DGC Ht          CGGYFTKPVKPLDLMLAVDELTAPAAE----------E----------------PLKVLV   269
WspR Pa         ANDYLVKLPDAIELVARIRYHSRSYIALQQ---RDE------------------------   148
PleD Cc         ASDFLTKPIDDVMLFARVRSLTRFKLVIDELRQREASGRRMGVIAGAAARLDGLGGRVLI   158
                ...::.*  . : *.  :   :

DGC Ht          VDDEPEAAAYHALLLEESGMLTHQVHH-PADALTVMERFSPDLLLVDVYMPVCSGEELAS   328
WspR Pa         ---------------------------------------------------AYRALR---   154
PleD Cc         VDDNERQAQRVA---AELGVEHRPVIESDPEKAKISAGGPVDLVIVNAAAKNFDGLRFTA   215
                                                      . .

DGC Ht          IIRQQPEHLGLPIIYLSSETDSQKQISAMSAGVEAFLTKPVQPEELVSAVQLRAE-----   383
WspR Pa         --------------------------------------------------------ESQQ   158
PleD Cc         ALRSEERTRQLPVLAMVDPDDRGRMVKALEIGVNDILSRPIDPQELSARVKTQIQRKRYT   275


DGC Ht          -----RLRLLRSLMTRDSMTGLYNHSTTTELINKNLAQAHRDNSQHAMAMIDIDHFKHVN   438
WspR Pa         QLLETNL-VLQRLMNSDGLTGLSNRRHFDEYLEMEWRRSLREQSQLSLLMIDVDYFKSYN   217
PleD Cc         DYLRNNLDHSLELAVTDQLTGLHNRRYMTGQLDSLVKRATLGGDPVSALLIDIDFFKKIN   335
                     .*     *   * :*** *:      ::    ::    .  :  :**:*.**  *

DGC Ht          DTHGHLAGDQVIITLARLLKS-RLRLSDIIGRYGGEEFVVLLKGINAEKAATLIDSLRED   497
WspR Pa         DTFGHVAGDEALRQVAGAIREGCSRSSDLAARYGGEEFAMVLPGTSPGGARLLAEKVRRT   277
PleD Cc         DTFGHDIGDEVLREFALRLAS-NVRAIDLPCRYGGEEFVVIMPDTALADALRIAERIRMH   394
                **.**  **:.:  .*  : .   *  *:  *******.::: .     *  : : :*

DGC Ht          FALIDFHT--GEVRFRCTFSAGISSFPAQ---PSTEPLRLSADQALYRAKHQGRNQVVIS   552
WspR Pa         VESLQISHDQPRPGSHLTVSIGVSTLVPGGGGQTFRVLIEMADQALYQAKNNGRNQVGLM   337
PleD Cc         VSGSPFTVAHGREMLNVTISIGVS--ATAGEGDTPEALLKRADEGVYQAKASGRNAVVGK   452
                .    :     .   . *.* *:*         : . *   **:.:*:** .*** *

DGC Ht          PELADDR---      559
WspR Pa         EQPVPPAPAG      347
PleD Cc         AA--------      454
```

Percent Identity Matrix - created by Clustal2.1

DGC *Ht*	100.00	28.30	27.94
WspR *Pa*	28.30	100.00	34.17
PleD *Cc*	27.94	34.17	100.00
