# Supplementary material for: Identification of two different chemosensory pathways in representatives of the genus Halomonas
Source: BMC Genomics. 2018 Apr 18;19:266. doi: 10.1186/s12864-018-4655-4 (PMC5907407; doi:10.1186/s12864-018-4655-4)
Supplement: Supplementary file 7 — Table S3. Protein percent identity matrices between the 16 microorganisms where clusters identical to cluster 2 were found. A) Percent identity matrix for diguanylate cyclase proteins from cluster 2-like sequences; B) Percent identity matrix for CheA proteins from cluster 2-like sequences; C) Percent identity matrix for CheR proteins from cluster 2-like. In blue: γ- Oceanospirillales, in red: α-Rhodospirillales, in green: γ- Chromatiales, in brown: γ- Enterobacterales. (PDF 61 kb) [file 12864_2018_4655_MOESM7_ESM.pdf]

A) DGCs

Percent Identity Matrix - created by Clustal2.1

	B.agres	C.young	H.boliv	**H.titKHS3**	H.titBH1	M.alkal	N.lacis	T.chlor	M.magne	M.maris	M.gryph	P.rhodo	M.marin	M.purpu	T.marin	A.vinos	T.drews
B.agres	100.00	70.50	39.38	39.56	39.49	41.13	40.62	35.69	39.11	39.00	39.52	38.91	37.34	36.45	37.03	38.11	37.36
C.young		100.00	40.70	40.15	40.07	40.84	39.67	36.79	39.70	39.78	40.85	40.11	36.73	36.82	37.59	39.78	38.11
H.boliv			100.00	91.77	91.59	55.22	53.32	42.20	45.14	44.49	44.65	43.48	43.67	42.93	42.03	44.73	41.74
H.titKHS3				100.00	99.82	54.14	52.42	42.39	44.04	43.57	44.65	44.02	42.75	42.75	41.49	43.45	41.20
H.titBH1					100.00	54.14	52.42	42.12	44.04	43.57	44.65	44.12	42.75	42.86	41.41	43.56	41.30
M.alkal						100.00	65.66	44.04	48.82	47.81	50.00	48.47	45.80	46.01	44.93	44.83	45.01
N.lacis							100.00	40.95	47.37	47.09	49.18	46.86	44.08	41.88	42.24	41.95	43.40
T.chlor								100.00	42.57	43.20	44.49	44.73	42.49	43.99	43.99	43.30	41.73
M.magne									100.00	81.11	64.40	58.14	47.43	43.64	46.00	47.09	47.45
M.maris										100.00	63.12	58.32	45.86	43.53	45.17	45.72	47.18
M.gryph											100.00	68.36	48.43	46.07	48.08	47.35	49.36
P.rhodo												100.00	47.80	44.52	46.56	47.56	48.38
M.marin													100.00	47.29	49.10	49.64	49.73
M.purpu														100.00	56.36	56.89	54.79
T.marin															100.00	57.96	59.55
A.vinos																100.00	67.44
T.drews																	100.00

B) CheAs

Percent Identity Matrix - created by Clustal2.1

	B.agres	C.young	H.boliv	H.titKHS3	H.titBH1	M.alkal	N.lacis	M.marin	T.chlor	P.rhodo	M.gryph	M.magne	M.maris	M.purpu	T.marin	A.vinos	T.drews
B.agres	100.00	74.18	46.34	46.78	46.49	45.45	46.77	46.38	43.84	45.19	48.91	48.92	48.63	42.14	44.49	47.37	47.95
C.young		100.00	45.89	46.47	46.19	44.99	46.60	45.78	42.71	44.36	47.51	48.40	48.26	43.45	44.02	48.06	47.50
H.boliv			100.00	90.84	91.13	58.99	60.61	49.06	54.20	52.89	55.10	54.49	54.05	46.68	49.14	50.65	51.37
H.titKHS3				100.00	99.58	59.32	60.34	48.65	53.74	53.56	55.36	55.32	54.56	46.30	48.16	50.64	50.43
H.titBH1					100.00	59.32	60.20	48.79	53.88	53.70	55.51	55.17	54.56	46.30	48.16	50.50	50.28
M.alkal						100.00	68.94	52.04	54.58	55.21	58.52	60.40	59.51	45.69	51.05	53.39	53.60
N.lacis							100.00	52.56	53.20	56.13	58.06	58.06	57.02	44.62	49.64	53.01	51.50
M.marin								100.00	49.58	52.94	58.06	57.12	57.20	49.23	55.54	53.69	55.20
T.chlor									100.00	53.82	55.17	57.02	56.12	46.43	48.68	51.76	50.42
P.rhodo										100.00	64.74	65.76	64.35	48.79	52.71	52.96	53.68
M.gryph											100.00	74.01	73.81	51.09	57.78	56.79	58.67
M.magne												100.00	87.39	50.36	56.08	56.96	58.59
M.maris													100.00	50.79	55.46	55.62	56.65
M.purpu														100.00	54.71	54.97	54.90
T.marin															100.00	63.32	64.31
A.vinos																100.00	71.39
T.drews																	100.00

C) CheRs

Percent Identity Matrix - created by Clustal2.1

	C.young	B.agres	M.marin	M.purpu	T.marin	T.drews	A.vinos	T.chlor	P.photo	M.gryph	M.magne	M.maris	H.boliv	H.titKHS3	H.titBH1	N.lacis	M.alkali
C.young	100.00	64.89	36.42	36.63	33.63	31.12	32.03	34.91	35.63	38.50	37.58	37.36	31.98	33.48	33.26	35.15	35.67
B.agres		100.00	35.76	36.38	35.46	32.62	34.20	35.47	35.62	40.31	38.43	37.99	31.47	33.18	32.74	35.06	36.01
M.marin			100.00	39.04	38.38	38.14	38.82	34.41	37.02	37.37	38.53	38.95	35.98	35.15	34.93	36.21	36.29
M.purpu				100.00	47.03	47.38	47.68	36.03	41.31	42.28	40.89	40.80	35.54	35.76	35.54	37.56	36.59
T.marin					100.00	50.91	49.90	36.80	39.22	39.22	38.63	37.77	35.52	34.74	34.52	35.13	37.04
T.drews						100.00	57.03	34.53	38.36	41.49	39.79	38.53	34.21	34.20	33.98	33.19	36.88
A.vinos							100.00	34.69	38.36	40.13	37.71	37.29	34.08	33.70	33.26	35.31	35.50
T.chlor								100.00	42.38	42.65	42.44	41.61	37.31	35.90	35.68	36.63	40.04
P.photo									100.00	51.70	49.58	49.16	36.89	35.81	35.59	38.78	38.78
M.gryph										100.00	55.67	56.30	38.75	38.02	37.58	39.92	41.19
M.magne											100.00	78.10	38.41	38.18	38.18	39.12	40.92
M.maris												100.00	38.11	36.15	36.36	37.66	39.67
H.boliv													100.00	81.78	81.99	40.99	40.04
H.titKHS3														100.00	99.17	41.23	40.42
H.titBH1															100.00	41.01	39.79
N.lacis																100.00	56.21
M.alkali																	100.00
